# Transcriptional Regulation of the Novel Theacrine Synthase Gene *CsTcS2* by the *CsTINY–CsWRKY33* Module Underpins Theacrine Biosynthesis in *Camellia sinensis*

**DOI:** 10.1111/pbi.70665

**Published:** 2026-04-15

**Authors:** Ting Wu, Lihua Zhu, Chenyu Shao, Siyi Xie, Na Li, Xinyu Li, Fang Wang, Lvwen Peng, Huiying Jin, Fanghuizi Shang, Jianan Huang, Zhonghua Liu, Na Tian, Shuoqian Liu

**Affiliations:** ^1^ State Key Laboratory of Tea Plant Germplasm Innovation and Resource Utilization Hunan Agricultural University Changsha China; ^2^ National Research Center of Engineering and Technology for Utilization of Botanical Functional Ingredients Changsha China; ^3^ Yuelushan Laboratory Changsha China; ^4^ Key Laboratory of Tea Science of Ministry of Education Hunan Agricultural University Changsha China; ^5^ Key Laboratory for Evaluation and Utilization of Gene Resources of Horticultural Crops, Ministry of Agriculture and Rural Affairs of China Hunan Agricultural University Changsha China

**Keywords:** alkaloid, AP2/ERF, tea plant, theacrine synthase, TINY, WRKY

## Abstract

Theacrine (1,3,7,9‐tetramethyluric acid) is a purine alkaloid detected in multiple wild and specialised tea germplasms (*Camellia sinensis*) from South China, including Kucha. However, the molecular mechanisms governing its biosynthesis remain poorly understood. Here, we identify CsTcS2 as a novel theacrine synthase in tea plant. Functional assays involving heterologous expression in *Nicotiana benthamiana*, antisense oligonucleotide‐mediated gene silencing, transient overexpression in tea plants and co‐expression with caffeine dehydrogenase (CsCDH) confirm its catalytic role in converting caffeine to theacrine. Transcription factors CsTINY and CsWRKY33 directly bind the CsTcS2 promoter and activate its transcription, as demonstrated by yeast one‐hybrid, dual‐luciferase reporter and electrophoretic mobility shift assays. Further molecular docking, yeast two‐hybrid, bimolecular fluorescence complementation, luciferase complementation, co‐immunoprecipitation and antisense inhibition experiments reveal a synergistic interaction between CsTINY and CsWRKY33 that regulates CsTcS2 expression and thus controls theacrine biosynthesis. Together, our findings unravel the transcriptional regulatory network underlying theacrine biosynthesis and provide a molecular foundation for breeding tea cultivars with elevated theacrine levels for health‐promoting applications.

## 1 Introduction

Tea plants ( *Camellia sinensis* ) produce several purine alkaloids that contribute to the stimulant effects and flavour profiles of tea infusions (Jin et al. 2014). Among them, caffeine (1,3,7‐trimethylxanthine) is the most abundant, typically accompanied by lower levels of theobromine (3,7‐dimethylxanthine) in most cultivated varieties (Ashihara et al. 2008). In contrast, theacrine (1,3,7,9‐tetramethyluric acid) is a structurally related, yet pharmacologically distinct, caffeine analogue. Theacrine differs from caffeine primarily by the addition of a ketone group at C_8_ and a methyl group at N_9_ (Wang et al. 2020; Zhang et al. 2020). This subtle structural divergence imparts unique physiological effects, exhibiting sedative (Xu et al. 2007) neuroprotective (Duan et al. 2020), antidepressant (Ouyang et al. 2021) and analgesic properties (Wang et al. 2010), without inducing the adverse side effects often associated with high caffeine intake, such as jitteriness, insomnia and increased intraocular pressure (Juliano and Griffiths 2004). These beneficial characteristics have spurred interest in developing low‐caffeine or caffeine‐free teas enriched in theacrine.

So far, several low‐caffeine germplasms have been discovered (Jia et al. 2024), such as *C*. *ptilophylla* (Ying et al. 2023), *C*. *gymnogyna* (Zhou et al. 2022), Hongyacha (Jin et al. 2018) and low‐caffeine hybrid tea plants (Wang, Liu, Wei, et al. 2023; Wang, Liu, Zhu, et al. 2023). However, theacrine accumulation is rare in cultivated tea cultivars and is predominantly found in a few wild accessions, notably *Kucha (Camellia sinensis*, Zheng et al. 2002). In *Kucha*, theacrine is mainly concentrated in young leaves (Ye et al. 2003). Theacrine biosynthesis proceeds via a specialised pathway beginning with hypoxanthine, which undergoes methylation and oxidation reactions through intermediates such as theobromine and caffeine (Wang et al. 2020; Zhang et al. 2020). Earlier studies utilising radiolabelled carbon (^14^C) demonstrated that theacrine biosynthesis is closely linked to the caffeine metabolic route (Zheng et al. 2002). Although several candidate genes involved in this pathway have been proposed (Chen et al. 2021; Li et al. 2021), none had been functionally validated until recently Zhang et al. (2020) successfully cloned *CkTcS*, an N_9_‐methyltransferase capable of converting 1,3,7‐trimethyluric acid into theacrine, confirming a key enzymatic step in the pathway. Similarly, Zhong et al. (2022) identified *TcS* as the causal gene responsible for theacrine accumulation through genetic mapping and demonstrated its activity in transgenic systems. These advances have effectively clarified the biosynthetic route of theacrine; however, the upstream regulatory mechanisms governing gene expression in this pathway remain unknown.

In plants, purine alkaloid biosynthesis is orchestrated by complex transcriptional networks, with transcription factors (TFs) playing critical regulatory roles (Li, He, et al. 2024; Zhang, Fu, et al. 2022). Pani and Mahapatra (2013) discovered that miR5021 targets 4‐hydroxy‐3‐methylbut‐2‐enyl diphosphate synthase and chloroplast terpenoid cyclase to regulate the biosynthesis of terpenoid indole alkaloids (TIA) in rose periwinkle. Jin et al. (2020) showed that miR902, miR1170 and miR1646 target *PMT*, *QPT* and *BBL*, respectively, thereby regulating nicotine biosynthesis. Chen et al. (2020) found that miR157b and miR160a target *HmSPL6* and cytochrome P450 genes (e.g., *CYP71A8*), influencing betacyanin biosynthesis in pitaya. Similarly, Shen et al. (2017) reported that cro‐miR164a/b, cro‐miR393d and cro‐novel‐92 may affect TIA biosynthesis via auxin signalling components (*CrARF10*, *CrARF16*, *CrARF17*). In tea, increasing evidence indicates that TFs are central regulators of metabolic pathways. In the caffeine pathway, the bHLH TF CsbHLH1 negatively regulates the expression of *TCS1* (caffeine synthase) by directly binding to its promoter. Downregulation of CsbHLH1 via miR1446a relieves this repression, resulting in elevated caffeine accumulation (Jin et al. 2024a). In addition, miR828a has been shown to cleave transcripts of the TF CsMYB114, thereby inhibiting theobromine biosynthesis (Jin et al. 2024b). More recently, Jiang et al. (2025) reported that Csi‐miR156e and Csi‐miR156k directly cleave *CsSPL6.1*, suggesting that this miRNA‐TF module may play a pivotal role in nitrogen assimilation.

Transcriptomic comparisons between *Kucha* and conventional tea cultivars have revealed differential expression of several TF families potentially involved in theacrine biosynthesis, with members of the NAC and HD‐ZIP families showing significant enrichment (Liu et al. 2022). Moreover, TFs from the AP2/ERF and WRKY families have been widely implicated in regulating secondary metabolism across diverse plant species.

The AP2/ERF superfamily, one of the largest plant‐specific TF families, plays pivotal roles in developmental regulation and stress responses, including alkaloid biosynthesis (Feng et al. 2020). Many AP2/ERF factors are responsive to jasmonate signalling and act as activators of key biosynthetic genes. For instance, in medicinal plants, MeJA‐induced expression of *EcAP2/ERFs* promotes the expression of genes such as *Ec6OMT* and *EcCYP719A5*, involved in benzylisoquinoline alkaloid biosynthesis (Yamada et al. 2020; Zhou and Memelink 2016). In tobacco, NtREF189 and NtERF199 coordinately activate genes in the nicotine biosynthetic pathway (Hayashi et al. 2020), while members of the ORCA subfamily (*ORCA1‐3*) in *Catharanthus roseus* regulate terpenoid indole alkaloid biosynthesis by binding to jasmonate‐responsive elements (Singh et al. 2020).

Similarly, WRKY TFs regulate alkaloid biosynthesis by binding to W‐box cis‐elements in target gene promoters (Li et al. 2022; Suttipanta et al. 2011; Wang et al. 2022; Zhang et al. 2023). In *Coptis japonica*, CjWRKY1 enhances the expression of genes involved in isoquinoline alkaloid production (Kato et al. 2007). In *Catharanthus roseus* , CrWRKY1 modulates terpenoid biosynthesis by targeting tryptophan decarboxylase (Suttipanta et al. 2011). OpWRKY6 in *Ophiorrhiza pumila* negatively regulates camptothecin biosynthesis by repressing multiple biosynthetic genes (Wang et al. 2022). In tropane alkaloid biosynthesis, AbWRKY1 and AbWRKY2 regulate key genes such as *PMT*, *CYP80F1* and *H6H* (Zhang et al. 2023). In lotus, *NnWRKY70a/b* directly activate benzylisoquinoline alkaloids pathway genes through W‐box binding (Li et al. 2022).

Despite mounting evidence of AP2/ERF and WRKY TFs as central regulators of alkaloid biosynthesis, their roles in theacrine biosynthesis remain largely unexplored. In this study, we investigated the molecular basis of theacrine production by functionally characterising *CsTcS2*, a key gene involved in its biosynthesis. We further elucidated the regulatory roles of CsTINY (AP2/ERF family) and CsWRKY33 (WRKY family) in modulating *CsTcS2* expression. Our findings provide important insights into the transcriptional regulation of theacrine biosynthesis and offer a solid foundation for the metabolic engineering and breeding of tea cultivars enriched in health‐promoting theacrine.

## 2 Results and Analysis

### 2.1 Genome‐Wide Screening and Identification of Theacrine Synthase

To elucidate the molecular mechanisms underlying the regulation of theacrine biosynthesis, we conducted homologous sequence alignments using the known theacrine synthase gene *CkTcS* (Genbank accession number: MN163831.1) against 3 individual tea reference genomes, including ‘Tieguanyin’ (Zhang et al. 2021), ‘Yunkang 10’ (Zhang et al. 2019) and ‘Shuchazao’ (Wei et al. 2018). We also queried the cultivar pangenome constructed from ‘Anjibaicha’, ‘Zijuan’ and ‘L168’ (Tariq et al. 2024). Analysis identified 3 homologous loci in the ‘Yunkang 10’ genome, all annotated as salicylic acid carboxyl methyltransferases (Data S1). Six homologous loci were identified in the ‘Shuchazao’ genome, with the locus CSS0032602, annotated as probable caffeine synthase 2, displaying the highest sequence identity (94%) to *CkTcS* (Data S1). The ‘Tieguanyin’ genome contained 10 homologous loci with annotations varying from caffeine synthase to jasmonate O‐methyltransferase and salicylic acid carboxyl methyltransferase. In the pangenome, we identified 7 CkTcS‐homologous sequences. Among them, 4 sequences, annotated as either probable caffeine synthase 2 or probable caffeine synthase 2 isoform X1, showed ≥ 94% but < 95.69% nucleotide identity to *CkTcS* (Data S1).

**FIGURE 1:**
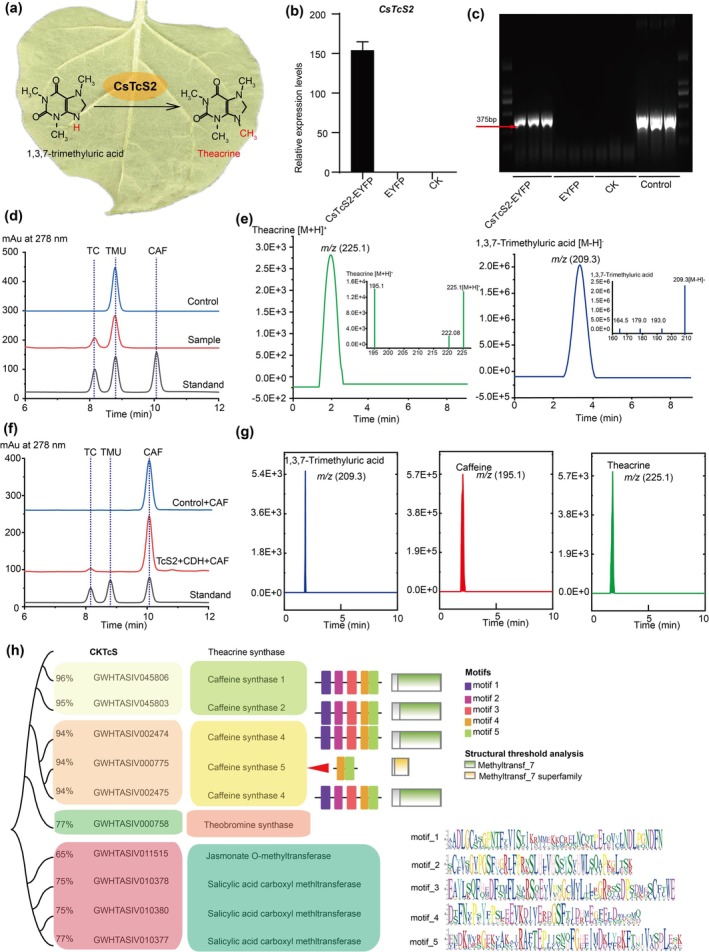
Heterologous expression of CsTcS2 in tobacco and production of theacrine from 1,3,7‐trimethyluric acid. (a) Schematic representation of theacrine biosynthesis catalysed by CsTcS2 upon injection of 1,3,7‐trimethyluric acid. (b) Relative expression levels of *CsTcS2* in tobacco leaves transiently expressing CsTcS2‐EYFP. (c) Electrophoresis analysis analysis confirming *CsTcS2* gene transient expression in tobacco leaves. Empty: Negative control; CK: Untreated control; Control: Positive control, the cloned *CsTcS2* gene plasmid. (d) HPLC analysis showing the production of theacrine (TC) from 1,3,7‐trimethyluric acid (TMU) in tobacco expressing CsTcS2. CAF: Caffeine; Standard: Authentic compound standards; Sample: CsTcS2‐expressing leaves injected with TMU; Control: Leaves expressing EYFP‐empty injected with TMU. (e) LC–MS analysis identifying theacrine (*m/z* 225.1) and 1,3,7‐trimethyluric acid (*m/z* 209.3) in tobacco extracts. (f) HPLC analysis of theacrine production from caffeine (CAF) after heterologous co‐expression of CsTcS2 and CsCDH in tobacco. Standard: Authentic standards; TcS2 + CDH + CAF: Leaves co‐expressing CsTcS2 and CsCDH with caffeine; CK + CAF: EYFP‐empty control leaves with caffeine. (g) LC–MS qualitative analysis confirming the presence of 1,3,7‐trimethyluric acid (blue, *m/z* 209.3), theacrine (green, *m/z* 225.1), and caffeine (red, *m/z* 195.1) in co‐expressed samples. (h) Phylogenetic tree, motif and structural threshold analysis.

However, these BLAST analyses (Data S1) indicate that none of the gene sequences currently available from tea plant genomes share greater than 97% identity with *CkTcS*, precluding a definitive identification based solely on sequence similarity. Therefore, candidate genes with sequence identities ≥ 94% were selected for functional characterisation using transient heterologous expression. Subsequent functional assays (Figure 1) revealed that among the candidates tested, the gene annotated as GWHTASIV000775 exhibited significant catalytic activity towards 1,3,7‐trimethyluric acid, the immediate biosynthetic precursor of theacrine, and was thus designated CsTcS2. Mapping to the genome assembly of ‘Tieguanyin’, the *CsTcS2* gene is located on chromosome Chr01 (GWHASIV00000001), spanning positions 43 757 717 to 43 775 343 on the negative strand (Figure S1). When tobacco leaves transiently expressing CsTcS2 were infiltrated with 1,3,7‐trimethyluric acid (Figure 1a–c), High‐Performance Liquid Chromatography (HPLC) analysis detected a prominent peak at a retention time of 8.1 min, coincident with authentic theacrine standards. This peak was notably absent in control leaves, clearly demonstrating the capability of CsTcS2 to catalyse the in vivo conversion of 1,3,7‐trimethyluric acid into theacrine (Figure 1d). Liquid Chromatograph Mass Spectrometry (LC–MS) analyses further supported this finding by detecting the characteristic theacrine ion at an *m/z* value of 225.1, matching previously published spectra and undetectable in the controls (Figure 1e). To fully verify the biosynthetic route from caffeine to theacrine, we co‐expressed CsTcS2 together with CsCDH, a caffeine dehydrogenase previously shown to catalyse caffeine oxidation into 1,3,7‐trimethyluric acid, in tobacco leaves (Zhu et al. 2025). Following exogenous caffeine administration, formation of theacrine was supported through both HPLC and LC–MS analyses, reinforcing the catalytic role of CsTcS2 in the theacrine biosynthesis pathway (Figure 1f,g; Figure S2). To compare the catalytic activity of our newly identified theacrine synthase, CsTcS2, with the previously reported CkTcS, we performed enzymatic kinetics assays of CsTcS2. The results (Figure S3a,b) showed that CsTcS2 exhibited a *K*_*cat*_ of 25.44 × 10^−3^ s^−1^ and a catalytic efficiency (*K*_*cat*_ */K*_*m*_) of 5.44 s^−1^ mM^−1^, which is higher than that reported for CkTcS, suggesting that CsTcS2 may possess stronger catalytic capacity.

### 2.2 Conserved Motif and Structural Feature Analysis of CsTcS2

To elucidate the evolutionary origin and structural specificity of CsTcS2, we performed a phylogenetic analysis using 10 gene loci homologous to CkTcS identified in the genome of *Camellia sinensis* var. ‘Tieguanyin’. These sequences clustered into four distinct clades, indicating considerable evolutionary divergence (Figure 1h). To explore its potential functional specialisation, we next compared the structural characteristics of CsTcS2 with other caffeine synthase‐like homologues known for methyltransferase activity. Motif composition analysis revealed that CsTcS2 contains only 2 conserved motifs, motifs 4 and 5, which differ substantially from the more complex motif architectures identified in other homologous proteins (Figure 1h). Additionally, *CsTcS2* is notably shorter, consisting of just 375 base pairs encoding a protein of 124 amino acids. Sequence alignments showed that this short protein aligns primarily with the catalytic C‐terminal region of the known CkTcS enzyme (Figure S4b). Subsequently, we compared the *CsTcS2* coding sequence across multiple cultivars and found it to be completely conserved, with no detectable sequence variation (Figure S4c; Data S7). Domain prediction analyses using InterProScan identified CsTcS2 as belonging to the methyltransf_7 superfamily (PFAM: PF03492), a group of S‐adenosyl‐L‐methionine (SAM)‐dependent methyltransferases (Figure S4a). This domain assignment was further corroborated by annotations from the CATH‐Gene3D and SSF (SCOP) databases, both confirming the presence of an intact SAM‐dependent methyltransferase domain. Collectively, these findings indicate that despite its minimal motif composition and reduced length, CsTcS2 retains all essential structural elements required for SAM‐mediated methylation (Figure S4a).

### 2.3 *CsTcS2* Transcript Levels Positively Correlate With Theacrine Accumulation in Tea Plant

**FIGURE 2:**
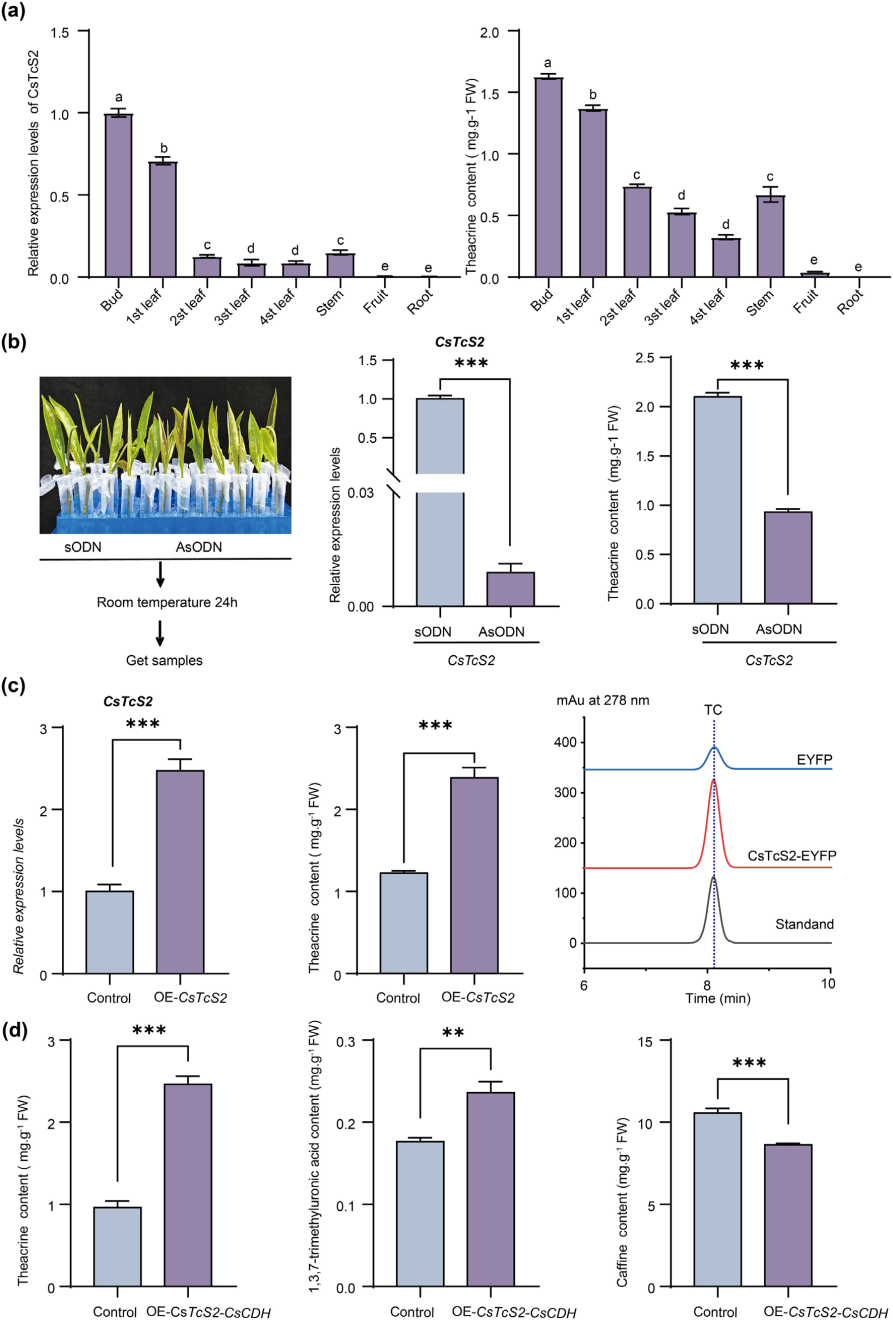
Expression analysis of *CsTcS2* in tea plants. (a) Tissue‐specific expression of *CsTcS2* and corresponding theacrine accumulation in cultivar ‘KC3’. (b) Effects of antisense oligonucleotide (AsODN‐*CsTcS2*) mediated *CsTcS2* suppression on theacrine content compared to scrambled control (sODN). (c) Increased theacrine accumulation upon *CsTcS2* transient overexpression. (d) Changes in caffeine, theacrine and 1,3,7‐trimethyluric acid contents upon transient co‐expression of *CsTcS2* and *CsCDH*. Different letters indicate significant differences (*p* < 0.05, Duncan's multiple range test). Statistical significance determined by two‐tailed Student's *t* tests (**p < 0.01, ***p < 0.001).

To determine the relationship between *CsTcS2* expression and theacrine accumulation, we first performed semi‐quantitative RT‐PCR using cDNA from representative Kucha and non‐Kucha accessions. A specific *CsTcS2* amplification band was detected only in the Kucha samples, whereas no corresponding band was observed in non‐Kucha cultivars (Figure S12), indicating that detectable theacrine accumulation is associated with the transcriptional deployment of *CsTcS2*. We then quantified transcript abundance and theacrine content in various tissues of the ‘KC3’ cultivar using quantitative real‐time polymerase chain reaction (qRT‐PCR) and HPLC analyses, respectively. The theacrine content varied substantially among tissues, ranging from 0.04 mg·g^−1^ to 1.63 mg·g^−1^ (Fresh weight, FW, Figure 2a). Notably, buds contained the highest theacrine concentration, followed by first leaves. In parallel, *CsTcS2* exhibited the highest expression levels in buds, followed closely by first leaves. Importantly, tissues with higher *CsTcS2* expression, particularly buds and the first leaves, also accumulated higher levels of theacrine (Figure 2a), supporting a positive association between *CsTcS2* transcript abundance and theacrine accumulation. To test whether efficient theacrine formation also depends on upstream precursor supply, we quantified the transcript level of *CsCDH* and the abundance of the theacrine precursor 1,3,7‐trimethyluric acid in representative Kucha versus non‐Kucha cultivars. Both *CsCDH* expression and precursor accumulation were significantly higher in the Kucha cultivars than in cultivars in which theacrine was undetectable (Figure S11), suggesting that enhanced upstream flux capacity may contribute to efficient theacrine formation.

To functionally validate this contribution of *CsTcS2* to theacrine accumulation, we conducted antisense oligonucleotide (AsODN)‐mediated silencing of *CsTcS2*, resulting in significantly decreased transcript levels and a corresponding reduction (~2.26‐fold) in theacrine accumulation (Figure 2b). Conversely, transient overexpression of *CsTcS2* significantly enhanced its transcript abundance and led to an increased accumulation (~1.95‐fold) of theacrine in tea plants (Figure 2c). Furthermore, co‐expression of *CsTcS2* and *CsCDH* resulted in markedly reduced caffeine levels, while simultaneously increasing the content of both theacrine and its direct precursor, 1,3,7‐trimethyluric acid (Figure 2d).

Together, these results support the conclusion that CsTcS2 functions as a novel enzyme in the theacrine biosynthetic pathway and that upstream precursor supply also contributes to efficient theacrine accumulation.

### 2.4 Screening and Identification of Transcription Factors Regulating *CsTcS2*

**FIGURE 3:**
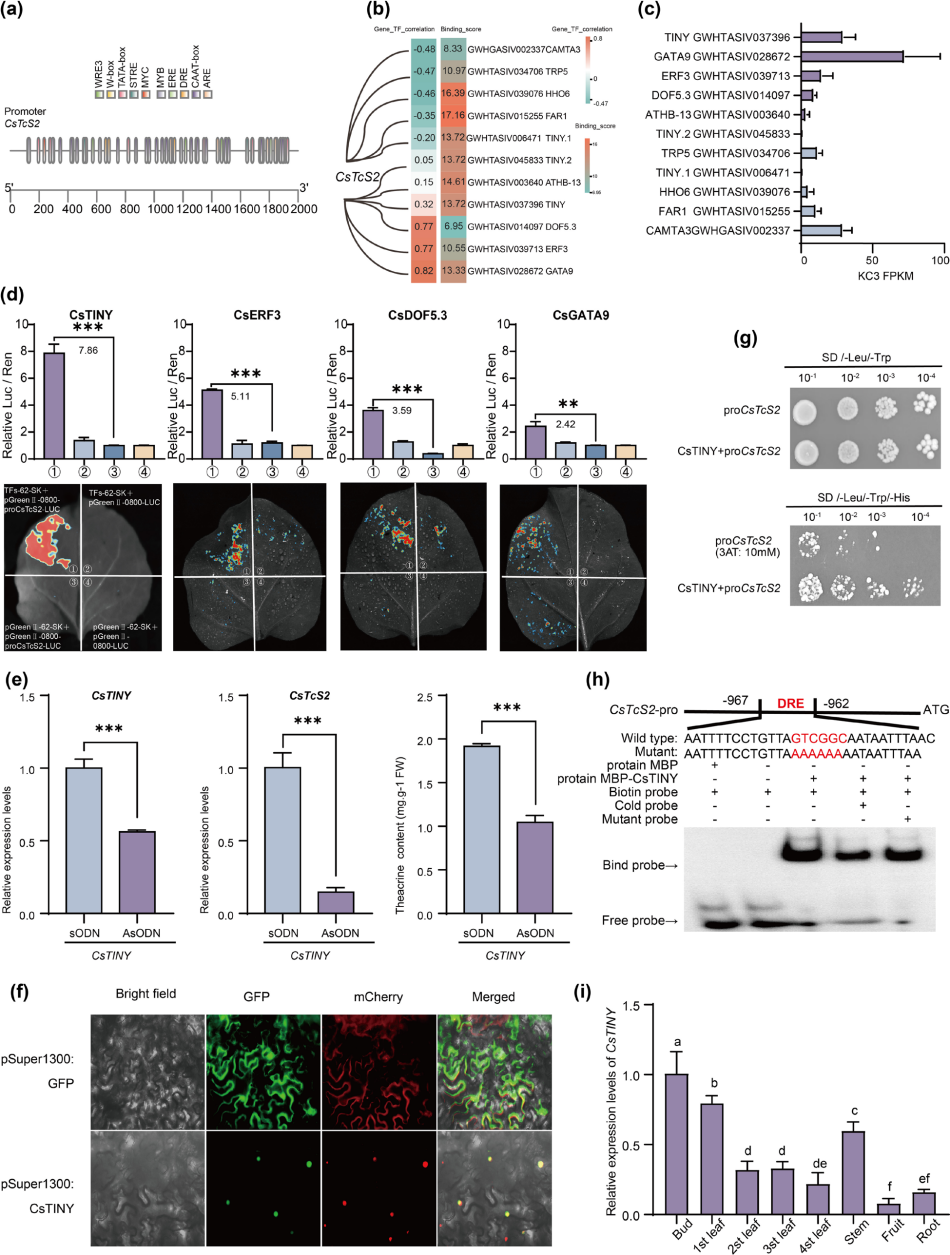
Identification of transcription factors regulating *CsTcS2*. (a) Analysis of cis‐regulatory elements in the *CsTcS2* promoter region. (b) Correlation analysis of candidate transcription factors predicted to regulate *CsTcS2*. (c) Expression levels of candidate transcription factors in the transcriptome of the high‐theacrine cultivar ‘KC3’. Purple represents the transcript abundance (FPKM) of candidate transcription factors predicted to be positively correlated, whereas light blue bars represent those predicted to be negatively correlated. (d) DLR assays quantifying transcriptional activation. (e) Reduction in *CsTcS2* expression and theacrine biosynthesis in CsTINY‐knockdown (AsODN‐CsTINY) leaves relative to controls (sODN‐CsTINY). Statistical significance determined by two‐tailed Student's t tests (***p < 0.001). (f) Subcellular localisation of CsTINY in tobacco leaves indicating nuclear localisation. (g) Y1H assay confirming CsTINY binding to the *CsTcS2* promoter; AD vector served as control. (h) EMSA demonstrating specific binding of CsTINY to the *CsTcS2* promoter. (i) Tissue‐specific expression of *CsTINY* in cultivar ‘KC3’.

To investigate the transcriptional regulation of *CsTcS2*, we first conducted an in‐depth analysis of its promoter region. Across 129 re‐sequenced accessions spanning the *CsTcS2* promoter (Data S7), we detected no major structural polymorphisms, with no large insertions or deletions observed (Figure S5). Then, we further isolated genomic DNA from the high‐theacrine cultivar ‘KC3’ to clone the *CsTcS2* promoter and analyse its cis‐regulatory elements. *CsTcS2* promoter analysis revealed multiple cis‐regulatory elements, including 4 WER3, 1 W‐box, 20 TATA‐box, 6 STRE, 10 MYC, 16 MYB, 2 ERE, 5 DRE, 43 CAAT‐box, 8 ARE (Figure 3a). The presence of these diverse regulatory motifs suggests complex transcriptional control of *CsTcS2* expression. Subsequently, genome‐wide TF predictions identified 11 TFs potentially regulating *CsTcS2*, comprising 6 positively correlated and 5 negatively correlated TFs (Figure 3b and Data S2). Transcriptomic data (Genbank accession number: PRJNA986690) from a previous ‘KC3’ study indicated that 4 positively correlated transcription factors, CsTINY (GWHTASIV037396), CsERF3 (GWHTASIV028672), CsGATA9 (GWHTASIV039713) and CsDOF5.3 (GWHTASIV014097), exhibited relatively high transcript abundance, whereas 4 negatively correlated transcription factors, namely CsTINY.1 (GWHTASIV006471), CsFAR1 (GWHTASIV015255), CsHHO6 (GWHTASIV039076) and CsTRP5 (GWHTASIV034706), displayed comparatively low expression levels (Figure 3c). To further validate these associations, we performed qRT‐PCR analysis to examine the expression levels of the 8 candidate transcription factors in leaves of Kucha and conventional cultivars. The results showed that *CsTINY* (*r* ≈ 0.86), *CsDOF5.3* (*r* ≈ 0.65), CsERF3 (*r* ≈ 0.78) and *CsGATA9* (*r* ≈ 0.76), were significantly correlated with *CsTcS2* expression, with *CsTINY* exhibiting the highest correlation (Figure S6). In contrast, the remaining predicted transcription factors, including *CsCAMTA3*, *CsTRP5*, *CsHHO6*, *CsFAR1* and *CsTINY.1*, showed weak correlations with *CsTcS2* expression (|*r*| < 0.55, Figure S6). Therefore, CsTINY, CsERF3, CsGATA9 and CsDOF5.3 were selected for subsequent experimental validation.

### 2.5 CsTINY Directly Binds the *CsTcS2* Promoter and Activates Its Transcription

To quantify the transactivation capacity of CsTINY, CsERF3, CsGATA9 and CsDOF5.3 towards the *CsTcS2* promoter, we performed dual‐luciferase reporter assays (DLR) in *N. benthamiana* leaves. Relative promoter activity was calculated as the firefly luciferase signal normalised to the Renilla luciferase internal control (LUC/REN) and expressed as fold change over the empty‐effector control. The results showed CsTINY produced the highest activation (7.86‐fold), whereas CsERF3, CsDOF5.3 and CsGATA9 showed progressively weaker activation (5.11‐fold, 3.59‐fold and 2.42‐fold, respectively; Figure 3d). To further evaluate the functional relevance of these four TFs in theacrine biosynthesis, we performed AsODN‐mediated gene silencing in leaves of ‘KC3’. Treatment with AsODN‐CsTINY significantly reduced *CsTINY* transcript levels relative to the scrambled oligonucleotide control (sODN‐CsTINY), which in turn led to a clear reduction in *CsTcS2* expression and a marked decrease in theacrine accumulation (Figure 3e). In contrast, silencing *CsERF3*, *CsDOF5.3*, or *CsGATA9* did not noticeably affect theacrine levels (data not shown). Therefore, CsTINY was selected as an upstream regulator of *CsTcS2* for further investigation.

Furthermore, genome‐wide predictions identified CsTINY, an AP2/ERF family member, as a high‐confidence candidate binding to the *CsTcS2* promoter with a high binding affinity score (score: 13.72, Figure 3b, Data S2 and Data S4). Structural modelling using AlphaFold 3 (Abramson et al. 2024) further supported this interaction, predicting a high‐confidence spatial association between the CsTINY protein and the *CsTcS2* promoter region (Figure S8a,b, Data S5).

To clarify the regulatory mechanism of CsTINY, we first examined its subcellular localisation. A pSuper1300‐CsTINY‐GFP fusion construct was transiently expressed in *N. benthamiana* epidermal cells, using the pSuper1300‐GFP vector as a negative control. Fluorescence microscopy confirmed that CsTINY localised exclusively to the nucleus (Figure 3f), where its signal overlapped with the nuclear marker, consistent with its role as a TF. Next, we performed a yeast one‐hybrid (Y1H) assay on selective medium containing 3‐AT to test whether CsTINY binds to the *CsTcS2* promoter and can drive reporter expression. In the control strain, almost no growth was observed at the 1000‐fold dilution, and no colonies were detectable at the 10 000‐fold dilution. By contrast, the CsTINY‐*CsTcS2* promoter strain still formed visible colonies even at the 10 000‐fold dilution, indicating that CsTINY binds specifically to the *CsTcS2* promoter (Figure 3g). To verify direct DNA‐protein interactions, electrophoretic mobility shift assays (EMSA) were performed using a purified CsTINY‐MBP fusion protein. The results showed that CsTINY specifically binds to the GTCGGC motif within the *CsTcS2* promoter region (Figure 3h). Furthermore, competition assays with an unlabelled probe resulted in a marked reduction of binding, confirming sequence‐specific interaction (Figure 3h; Figure S9).

To further evaluate the regulatory role of CsTINY in theacrine biosynthesis, we examined its transcript levels across different tissues of the ‘KC3’ cultivar using qRT‐PCR. The results showed *CsTINY* was expressed in all tested tissues, with significantly higher expression observed in buds and the first leaves (Figure 3i). This expression pattern closely matched that of *CsTcS2* and showed a significant positive correlation between *CsTINY* and *CsTcS2* (*r* = 0.93; Figure 2a; Figure 3i; Figure S7). Collectively, these findings identify CsTINY as a transcription factor of *CsTcS2*, directly binding its promoter and activating its transcription to promote theacrine biosynthesis in tea plants.

### 2.6 Identification and Validation of CsWRKY33 as a CsTINY Interacting Protein

**FIGURE 4:**
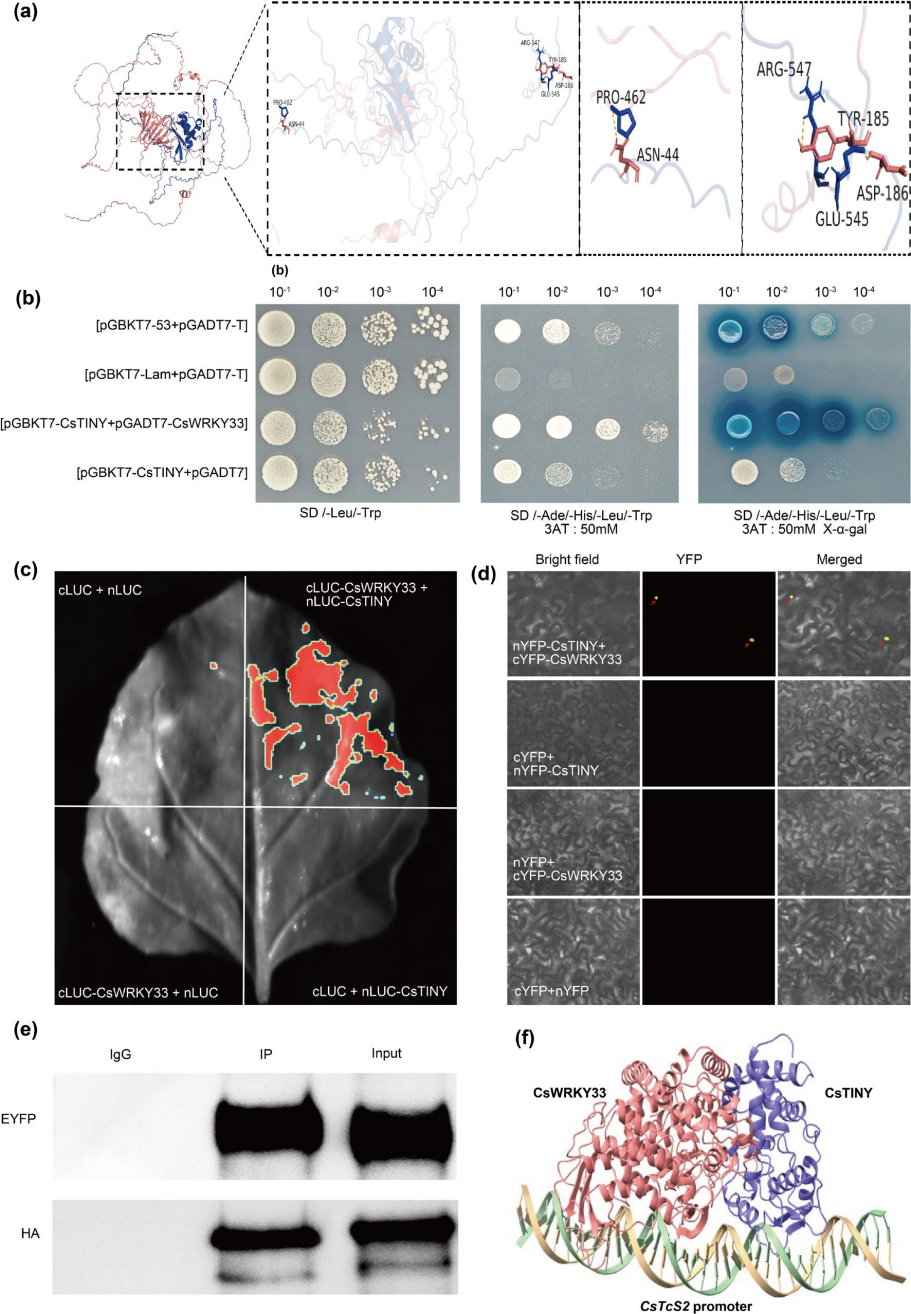
Identification and validation of CsWRKY33 as a CsTINY‐interacting protein. (a) Molecular docking of CsTINY (blue) and CsWRKY33 (pink). Yellow dashed lines represent hydrogen bonds. (b) Y2H assay validating CsTINY‐CsWRKY33 interaction. Positive control: PGBKT7‐53 + pGADT7‐T; negative control: PGBKT7‐Lam + pGADT7‐T. (c) LCA demonstrating in vivo interaction between CsTINY and CsWRKY33. (d) BiFC confirming CsTINY‐CsWRKY33 interaction localised to the nucleus. (e) Co‐immunoprecipitation (Co‐IP) analysis showing the interaction between CsWRKY33 and CsTINY. Protein extracts from *N. benthamiana* leaves co‐expressing CsWRKY33‐EYFP and CsTINY‐HA were subjected to immunoprecipitation using anti‐GFP antibodies. The precipitated complexes were detected by immunoblotting with anti‐GFP and anti‐HA antibodies, respectively. EYFP and HA signals were detected in both the input and IP lanes but not in the IgG control, confirming the in vivo interaction between CsWRKY33 and CsTINY. (f) Schematic model depicting the binding of CsTINY and CsWRKY33 to the *CsTcS2* promoter region.

To identify proteins interacting with CsTINY, we conducted a yeast two‐hybrid (Y2H) screen using CsTINY as bait against a tea cDNA library. This approach identified CsWRKY33 as a potential interacting partner of CsTINY (Figure S10 and Data S6). To structurally validate this interaction, molecular docking analysis was performed. Docking simulations indicated a stable interaction between CsTINY (blue) and CsWRKY33 (pink), mediated by multiple hydrogen bonds (yellow dashed lines, Figure 4a). Specifically, residues PRO462, ARG547 and GLU545 of CsTINY formed predicted hydrogen bonds with residues ASN44, TYR185 and ASP186 of CsWRKY33, respectively. The calculated binding energy of −209.3 kcal/mol suggested a highly specific and stable complex formation. Experimental validation using Y2H assays confirmed this protein–protein interaction, as yeast cells co‐transformed with CsTINY and CsWRKY33 constructs grew robustly on stringent selective medium (SD/−Ade/−His/−Leu/−Trp) (Figure 4b).

We further validated the interaction in planta using luciferase complementation assays (LCA) in *N. benthamiana* leaves. Strong luminescence signals were detected exclusively in leaves co‐expressing CsWRKY33‐cLUC and CsTINY‐nLUC, whereas no signals appeared in negative controls (Figure 4c). Consistent results were obtained from bimolecular fluorescence complementation (BiFC) assays, wherein co‐expression of CsWRKY33‐cYFP and CsTINY‐nYFP restored YFP fluorescence specifically in the nucleus (Figure 4d), confirming physiological relevance. Moreover, co‐immunoprecipitation (Co‐IP) showed that HA‐tagged CsWRKY33 co‐precipitated with anti‐GFP antibodies targeting CsTINY‐EYFP, and that CsTINY‐EYFP was reciprocally recovered with anti‐HA antibodies (Figure 4e), thereby supporting an in vivo association between CsWRKY33 and CsTINY. Collectively, these complementary structural and experimental approaches demonstrate that CsWRKY33 directly interacts with CsTINY, implicating a coordinated regulatory role in controlling theacrine biosynthesis.

### 2.7 CsWRKY33 Directly Binds the *CsTcS2* Promoter and Activates Its Transcription

**FIGURE 5:**
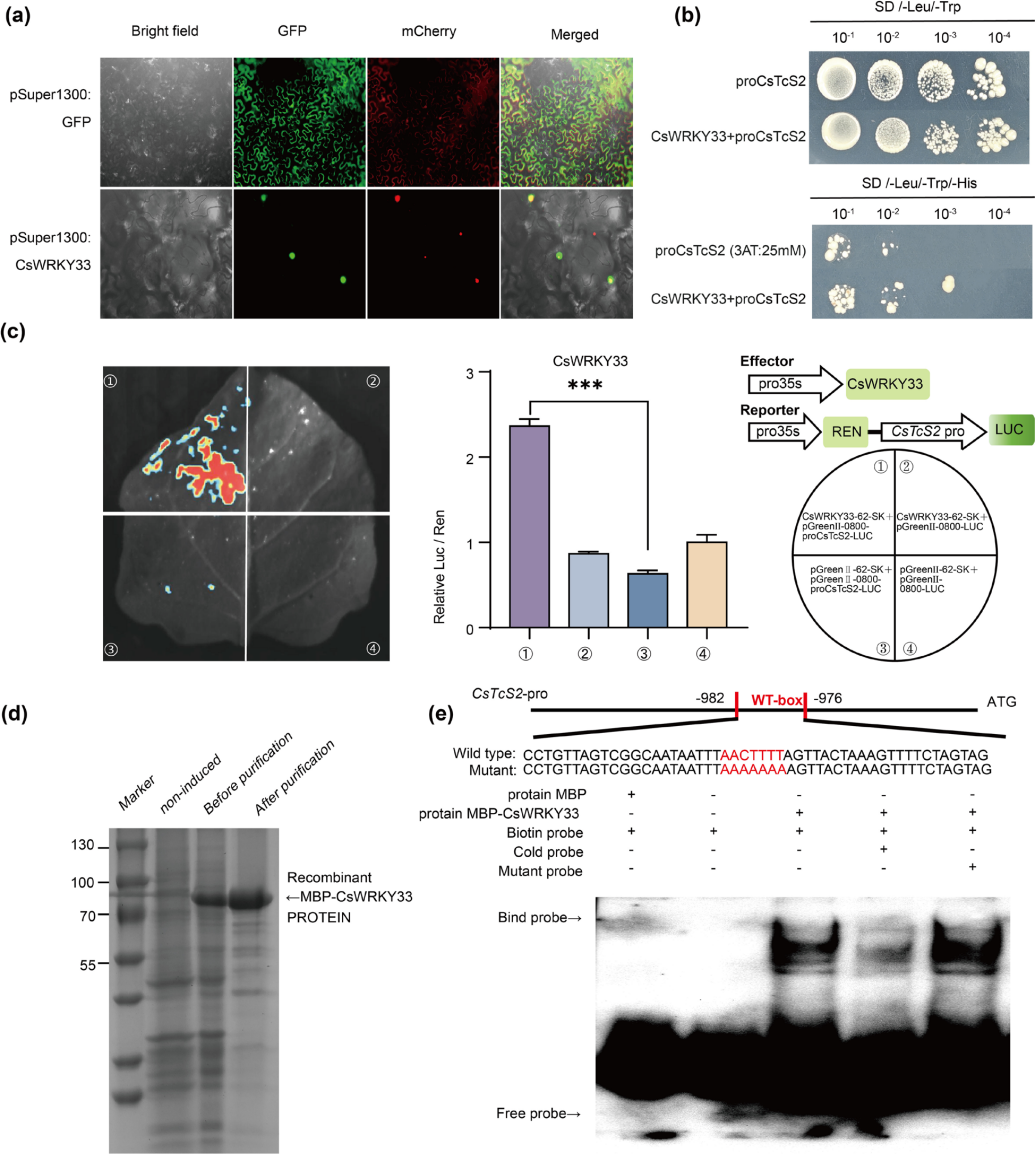
CsWRKY33 directly binds and activates the *CsTcS2* promoter. (a) Nuclear localisation of CsWRKY33‐GFP fusion protein in tobacco leaf epidermal cells. (b) Y1H assay confirming CsWRKY33 binding and activation of the *CsTcS2* promoter. (c) Effector and reporter constructs used in DLR assay. DLR assay validating transcriptional activation of the *CsTcS2* promoter by CsWRKY33 in *N. benthamiana* leaves (***p < 0.001, two‐tailed Student's t test). (d) SDS‐PAGE analysis of purified MBP‐CsWRKY33 fusion protein. (e) EMSA demonstrating direct binding of CsWRKY33 to the *CsTcS2* promoter region.

To investigate whether CsWRKY33 directly regulates *CsTcS2* expression, we first employed structural modelling using AlphaFold 3, which predicted a close spatial association between CsWRKY33 and CsTINY at the *CsTcS2* promoter region, suggesting the potential formation of a transcriptional regulatory complex (Figure 4f; Figure S8c and Data S5). Subsequent subcellular localisation analysis confirmed CsWRKY33 as a nuclear‐localised protein. Transient expression of a pSuper1300‐CsWRKY33‐GFP fusion construct in *N. benthamiana* epidermal cells revealed that the GFP signal colocalised with a nuclear mCherry marker, indicating exclusive nuclear localisation and supporting its proposed function as a TF (Figure 5a).

Y1H assays were performed to assess whether CsWRKY33 binds to and activates the *CsTcS2* promoter. On triple‐dropout plates, the control strain showed no growth at the 1000‐fold dilution, whereas the CsWRKY33‐*CsTcS2* promoter strain still formed visible colonies at the same dilution (Figure 5b). These results indicate a specific interaction between CsWRKY33 and the *CsTcS2* promoter and indicate that CsWRKY33 can drive expression of the *CsTcS2* promoter‐driven LUC reporter, resulting in a significant 2.36‐fold increase in the LUC/REN ratio compared with the control, verifying its transactivation capability (Figure 5c).

Finally, direct DNA‐protein interactions were confirmed by EMSA using a biotin‐labelled probe corresponding to the AACTTTT motif in the *CsTcS2* promoter region. A distinct protein‐DNA complex was observed, and this shifted band was effectively abolished by competition with unlabelled probe, demonstrating specificity of binding (Figure 5d,e). Collectively, these data establish CsWRKY33 as a nuclear TF that directly binds to and activates the *CsTcS2* promoter, underscoring its pivotal role in the transcriptional regulation of theacrine biosynthesis in tea plants.

### 2.8 CsTINY Activates CsWRKY33 and Cooperates It to Promote *CsTcS2* Transcription and Theacrine Biosynthesis

**FIGURE 6:**
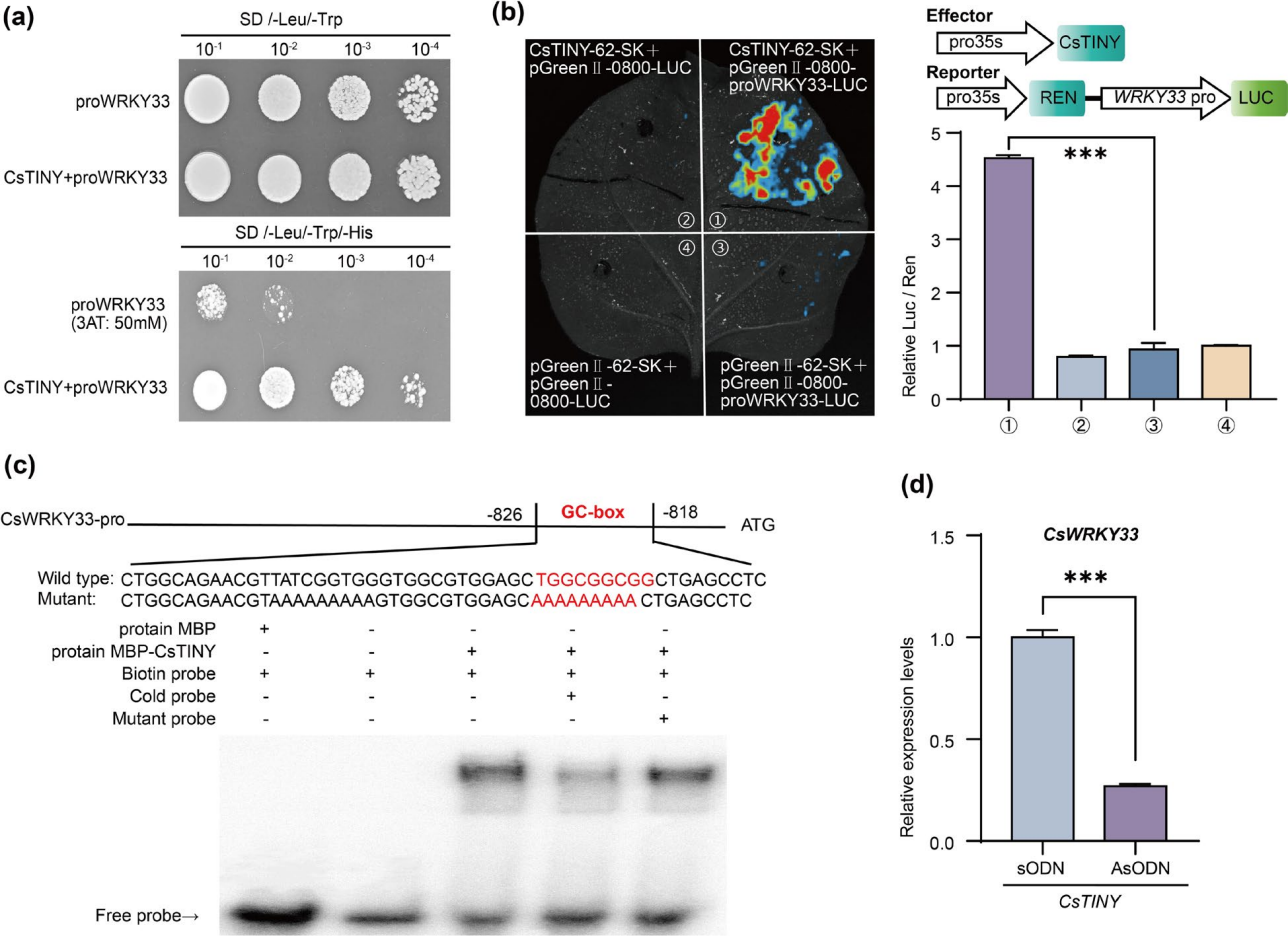
CsTINY enhances the regulatory effect of CsWRKY33 on *CsTcS2*. (a) Y1H assay confirming CsTINY binding and activation of the *CsWRKY33* promoter. (b) Efector and reporter constructs used in DLR assay. DLR assay validating transcriptional activation of the *CsWRKY33* promoter by CsTINY in *N. benthamiana* leaves. (c) EMSA demonstrating direct binding of CsTINY to the *CsWRKY33* promoter region. (d) The expression of *CsWRKY33* decreased after silencing the *CsTINY* gene.

**FIGURE 7:**
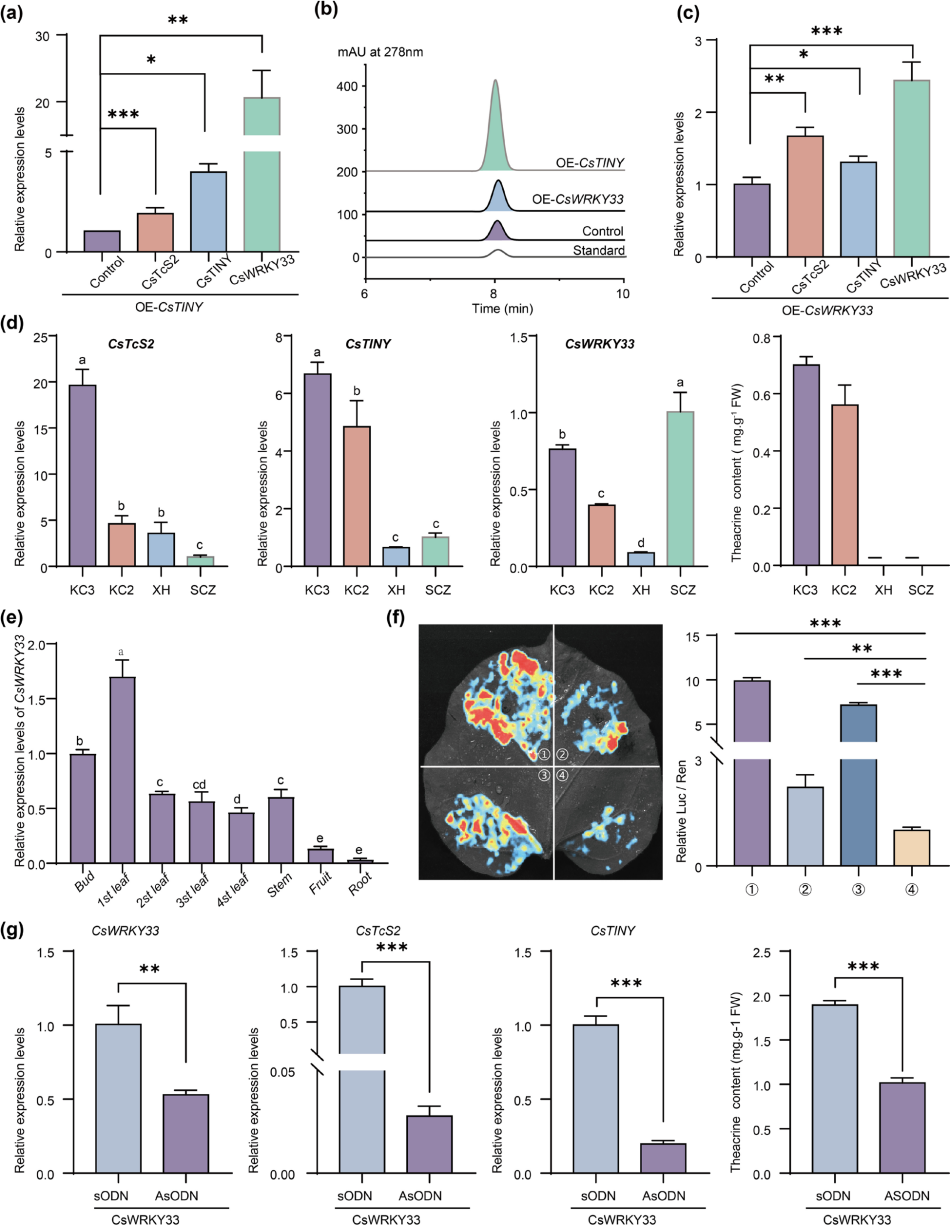
CsTINY‐CsWRKY33 module synergistically regulates *CsTcS2* transcription and theacrine biosynthesis. (a) Expression levels of *CsTcS2*, *CsTINY* and *CsWRKY33* increased compared with the control group, after transient overexpression of *CsTINY*. (b) Theacrine content in *CsTINY* and *CsWRKY33* overexpressing tea plants determined by HPLC. (c) After transient overexpression of *CsWRKY33*, the expression of *CsTcS2*, *CsTINY* and *CsWRKY33* increased compared with the control group. (d) Expression levels of *CsWRKY33*, *CsTINY and CsTcS2* across different Kucha cultivars and theacrine, with cultivar ‘SCZ’ as control. Different letters indicate significant differences. (e) Tissue‐specific expression of *CsWRKY33* in cultivar ‘KC3’. (f) DLR assay showing synergistic activation of the *CsTcS2* promoter by co‐expression of CsTINY and CsWRKY33 in *N. benthamiana* leaves (g) Reduced expression of *CsTINY* and *CsTcS2* and decreased theacrine biosynthesis in CsWRKY33‐knockdown (AsODN‐CsWRKY33) leaves relative to controls (sODN‐CsWRKY33). (*p* < 0.05, Duncan's multiple range test; **p < 0.01, ***p < 0.001, two‐tailed Student's *t* test).

To determine whether CsTINY regulates the transcription of *CsWRKY33*, we first performed a Y1H assay. The results showed that CsTINY bound the *CsWRKY33* promoter and significantly activated the promoter‐driven reporter gene in yeast (Figure 6a). Consistently, a DLR assay in *N. benthamiana* demonstrated that co‐expression of CsTINY with a luciferase reporter under the control of the *CsWRKY33* promoter led to a 4.52‐fold increase in the LUC/REN ratio compared with the control, confirming transcriptional activation of the *CsWRKY33* promoter by CsTINY (Figure 6b). We next assessed direct DNA‐protein binding. EMSA using a biotin‐labelled probe containing the TGGCGGCGG motif from the *CsWRKY33* promoter revealed a specific protein‐DNA complex. This shifted complex was effectively competed by unlabelled probe, demonstrating sequence‐specific binding of CsTINY to the *CsWRKY33* promoter region (Figure 6c). We then examined this regulatory relationship in planta. AsODN‐mediated suppression of CsTINY in tea leaves significantly reduced *CsWRKY33* transcript levels (Figure 6d), whereas transient overexpression of CsTINY led to significantly higher *CsWRKY33* expression (Figure 7a,b). Taken together, these results indicate that CsTINY functions as an upstream transcriptional regulator of *CsWRKY33* by directly binding its promoter and activating its transcription.

We next investigated whether CsWRKY33 and CsTINY act together to regulate *CsTcS2* expression and theacrine biosynthesis. The expression pattern analysis of *CsWRKY33* across different Kucha tea cultivars was performed by qRT‐PCR using the non‐Kucha cultivars ‘SCZ’ as a control. And the results showed that *CsWRKY33* transcripts were detected in all tested cultivars (Figure 7d). Among the Kucha cultivars, *CsWRKY33* expression level was highest in ‘KC3’, a high‐theacrine cultivar, followed by ‘KC2’ (Figure 7d). Notably, *CsWRKY33* expression was also relatively high in some non‐Kucha cultivars (Figure 7d), whereas CsTINY remained low (Figure 7d), suggesting that CsWRKY33 alone is insufficient to robustly activate *CsTcS2* and likely requires CsTINY co‐expression for effective induction. Moreover, tissue profiling in ‘KC3’ showed that *CsWRKY33* accumulated preferentially in buds and young leaves, resembling the expression patterns of *CsTINY* and *CsTcS2* and matching the tissue distribution of theacrine accumulation (Figure 7e). Across tissues, the transcript levels of *CsWRKY33* (*r* ≈ 0.88), *CsTINY* (*r* ≈ 0.96) and *CsTcS2* (*r* ≈ 0.94) were each significantly correlated with theacrine content (Figure 2a; Figure 3i; Figure 7e; Figure S7).

To test synergistic activation of CsWRKY33 and CsTINY at the promoter level, DLR assays were conducted in *N. benthamiana* leaves. Co‐expression of CsWRKY33 with CsTINY significantly enhanced the *CsTcS2* promoter‐driven reporter activity compared to CsTINY alone. Specifically, CsTINY alone increased LUC/REN reporter activity by 7.86‐fold, and CsWRKY33 alone increased it by 2.21‐fold, whereas co‐expression of CsWRKY33 further augmented this activation to 9.93‐fold, demonstrating that CsWRKY33 enhances CsTINY‐mediated transcriptional activation of the *CsTcS2* promoter (Figure 7f).

**FIGURE 8:**
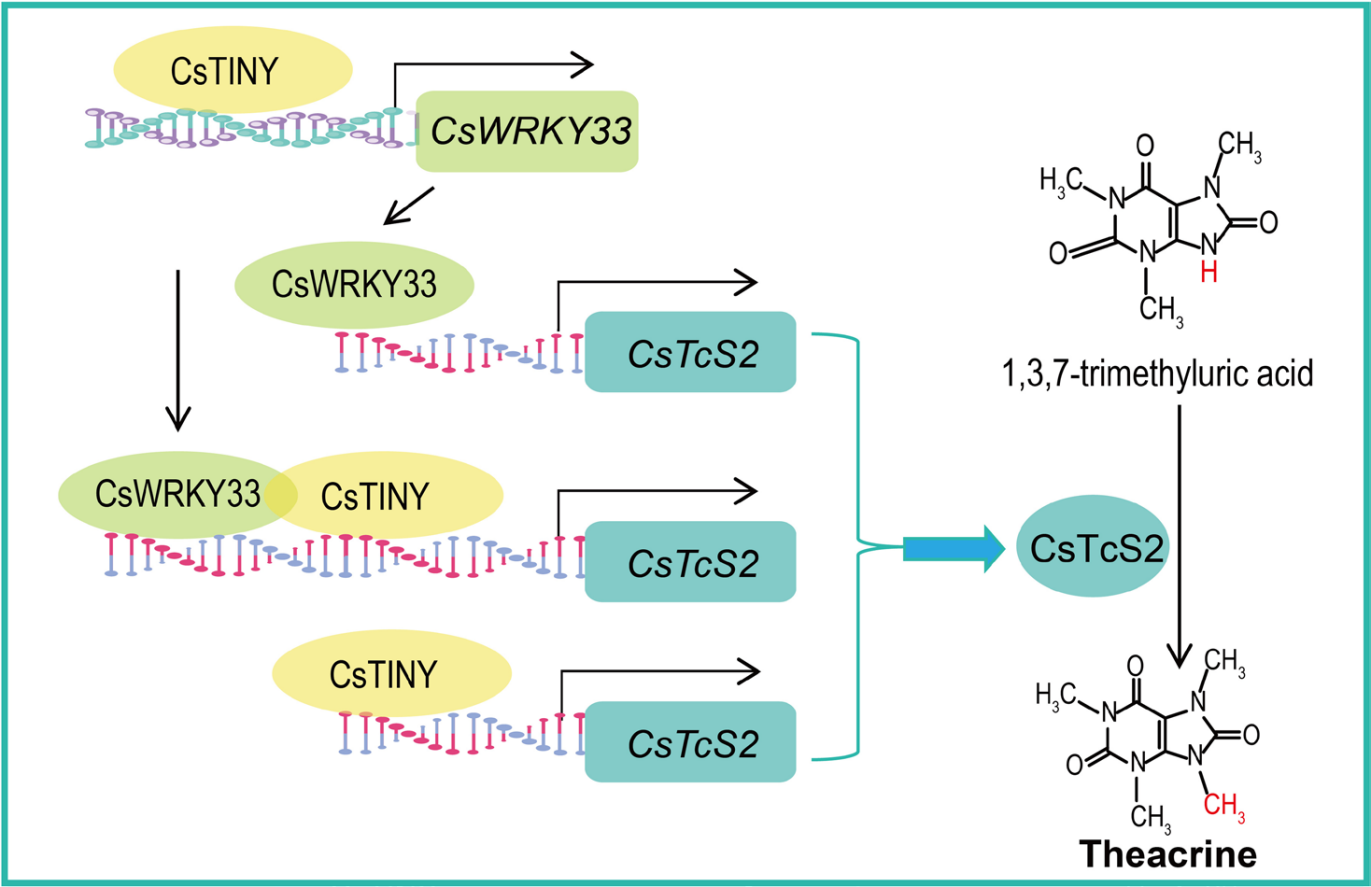
Proposed working model of CsTINY‐CsWRKY33‐mediated regulation of *CsTcS2* expression and theacrine biosynthesis. CsTINY and CsWRKY33 cooperatively activate *CsTcS2* transcription, leading to enhanced biosynthesis of theacrine in tea plants.

To further assess the functional relevance of this regulatory module, AsODN‐mediated gene silencing was employed. Treatment of tea leaves with AsODN‐CsWRKY33 significantly suppressed *CsWRKY33* expression compared to the scrambled oligonucleotide control (sODN‐CsWRKY33). Consequently, expression levels of *CsTINY* and *CsTcS2* were also significantly reduced, along with a marked decrease in theacrine content (Figure 7g). Meanwhile, transient overexpression of *CsWRKY33* in tea plant led to increased expression of *CsTINY* and *CsTcS2*, accompanied by an elevated theacrine content (Figure 7b,c). These findings indicate that CsWRKY33 not only directly regulates *CsTcS2* transcription but also cooperates with CsTINY to synergistically modulate theacrine biosynthesis in tea plants (Figure 8).

## 3 Discussion

Theacrine is a purine alkaloid predominantly found in select cultivars of tea, notably in the Kucha, and exhibits a wide spectrum of beneficial biological activities, including improved sleep quality (Xu et al. 2007), enhanced lipid metabolism (Lv et al. 2022) and potent antioxidant properties (Li et al. 2013). These effects render theacrine a promising compound for nutraceutical and therapeutic applications. However, its natural distribution is limited to a few tea cultivars, and even within these, theacrine typically accumulates at low levels (Wang et al. 2010). This scarcity imposes substantial economic and technical challenges for large‐scale extraction, thereby restricting its widespread commercial use. Developing tea cultivars with enhanced theacrine content offers a promising strategy to produce value‐added functional tea products. In this context, genome editing technologies, such as CRISPR/Cas9 (Zhong et al. 2022), provide powerful tools to engineer metabolic pathways. However, these tools require a detailed understanding of theacrine biosynthesis and its regulatory networks, which until now have remained largely unexplored. The present work defines CsTcS2 as a functional theacrine synthase and proposes a CsTINY‐CsWRKY33‐*CsTcS2* regulatory module controlling theacrine biosynthesis (Figure 8). CsTINY and CsWRKY33 activate *CsTcS2* directly via promoter binding and synergistically via protein interaction, while CsTINY additionally upregulates *CsWRKY33* to reinforce *CsTcS2* expression. This regulatory module provides molecular targets for future cultivar improvement.

### 3.1 Novel Theacrine Synthase Gene Was Mapped in the Tea Plant Genome

Although the theacrine synthase gene *CkTcS* has previously been reported in Kucha (Zhang et al. 2020), its genomic position could not be confidently assigned in widely used *C. sinensis* reference genomes, which impeded analysis of its native promoter, allelic variation and regulatory context. Our genome‐wide screen across multiple genomes of ‘Tieguanyin’ (Zhang et al. 2021), ‘Yunkang 10’ (Zhang et al. 2019) and ‘Shuchazao’ (Wei et al. 2018) and a pangenome incorporating ‘Anjibaicha’, ‘Zijuan’ and the wild accession ‘L168’ (Tariq et al. 2024) did not recover any homologue with > 97% nucleotide identity to the reported *CkTcS* sequence (Zhang et al. 2020) (Data S1). This suggested that the previously described *CkTcS* is either absent from, structurally diverged in, or not assembled in these references, and therefore could not serve as an experimentally tractable genomic target for regulatory dissection or breeding.

In contrast, *CsTcS2* was cloned from the ‘KC3’ cultivar and precisely localised to Chr01 (43757717‐43 775 343 bp; Figure S1). This provides a theacrine synthase gene with a confirmed chromosomal position in tea. Positioning *CsTcS2* in the genome is not only a mapping exercise; it defines a manipulable locus whose promoter, chromatin context and allelic diversity can now be interrogated directly. From a genetic improvement perspective, having a physically anchored biosynthetic gene is a prerequisite for cis‐regulatory editing, development of expression‐based markers for the selection and introgression of favourable alleles into commercial backgrounds.

The enzymatic features of CsTcS2 suggest functional specialisation within purine alkaloid metabolism. CsTcS2 encodes a 124‐amino acid protein that retains the core SAM‐dependent methyltransferase domain (PF03492; methyltransf_7 superfamily) despite being structurally compact (Figure 1h; Figure S4a). Phylogenetic placement indicates divergence from canonical caffeine synthase‐like enzymes (Figure 1h, Chen et al. 2021), implying that CsTcS2 represents a specialised branch of SAM‐dependent N‐methyltransferases acting at the terminal step of theacrine biosynthesis. Its catalytic profile is quantitatively distinct: CsTcS2 exhibits higher theacrine‐forming activity than CkTcS (~2.22‐fold; Figure S3a,b), supporting the view that CsTcS2 is not only structurally adapted but kinetically optimised for converting 1,3,7‐trimethyluric acid to theacrine (Figure 1a–e, Figure 2b,c). This is consistent with a recurring theme in plant specialised metabolism, in which pathway end‐point enzymes evolve enhanced catalytic efficiency towards lineage‐characteristic metabolites (Bouillé et al. 2025).

The biochemical role of CsTcS2 has broader relevance for purine alkaloid composition in tea. Conventional tea cultivars tend to accumulate caffeine as the major xanthine alkaloid (Jia et al. 2024), whereas rare low‐caffeine or caffeine‐poor cultivars, such as Hongyacha (Jin et al. 2018), cocoa tea, *C*. *gymnogyna* (Teng et al. 2020) and low‐caffeine hybrids (Wang, Liu, Wei, et al. 2023; Wang, Liu, Zhu, et al. 2023) often accumulate theobromine as the major alternative purine alkaloid. However, these low‐caffeine germplasm resources generally show weaker field performance, including reduced adaptability and stress tolerance, and thus have not been widely deployed in large‐scale cultivation. By contrast, Kucha‐type germplasm combines relatively good environmental adaptability with high theacrine accumulation, and theacrine itself has been associated with sleep‐related benefits (Xu et al. 2007). In this context, redirecting caffeine‐derived intermediates into theacrine via CsTcS2 represents a mechanistically distinct strategy compared with simply suppressing caffeine biosynthesis. A strict loss‐of‐function approach that blocks caffeine biosynthesis can lower total purine alkaloid output, which may negatively impact plant defence and growth. By contrast, CsTcS2 enables a flux‐redistribution model in which caffeine is enzymatically converted by CsCDH (Zhu et al. 2025) to 1,3,7‐trimethyluric acid and subsequently methylated by CsTcS2 to produce theacrine (Figure 1f,g, Figure 2d, and Data S2). This metabolic rerouting suggests that high‐theacrine with low‐caffeine chemotypes could be achieved by channelling purine alkaloid flux towards theacrine rather than by silencing the pathway.

Interestingly, coding sequence comparison of *CsTcS2* across cultivars (Figure S4c) revealed no disruptive sequence variation between Kucha‐type and non‐Kucha cultivars, and key upstream enzymes such as CsCDH are present in both (Zhang et al. 2020; Zhu et al. 2025). Nonetheless, substantial theacrine accumulation is largely restricted to Kucha cultivars (Figure 7d). This indicates that the biosynthetic capacity for theacrine is not a unique structural feature of Kucha cultivars; instead, the main source of variation is likely regulatory. In other words, most *C. sinensis* cultivars encode the capacity to synthesise theacrine but do not transcriptionally deploy that capacity at levels sufficient to generate high theacrine content. The observation that *CsTcS2* transcript abundance is positively correlated with theacrine content among tissues in ‘KC3’, with buds showing the highest *CsTcS2* expression and the highest theacrine accumulation, followed by the first leaves (Figure 2a), indicates that transcriptional regulation at the *CsTcS2* promoter is an important determinant of pathway flux, which shifts the mechanistic focus from enzyme presence or absence to transcriptional activation. Although *CsTcS2* transcript levels were comparable between the Kucha cultivar ‘KC2’ and ‘XH’, detectable theacrine was observed only in ‘KC2’ (Figure 7d). This mismatch is consistent with a supply‐limited regime in ‘XH’: both the transcript level of the upstream enzyme CsCDH and the abundance of the immediate precursor 1,3,7‐trimethyluric acid were significantly higher in ‘KC2’ than in ‘XH’ (Figure S11), suggesting that precursor availability and upstream flux capacity can gate theacrine output even when *CsTcS2* transcripts are present. Besides, post‐transcriptional or post‐translational regulation may also contribute to this process (Cheng et al. 2024).

### 3.2 CsTINY Regulates *CsTcS2* Expression to Improve Theacrine Accumulation

The *CsTcS2* promoter is enriched in cis‐regulatory elements (e.g., WER3, W‐box, TATA‐box, STRE, MYC/MYB, ERE, DRE, CAAT‐box and ARE; Figure 3a), suggesting regulation by TF families that couple developmental cues with abiotic/biotic stress responses, including AP2/ERF and WRKY (Chen et al. 2021; Zhang, Jin, et al. 2022; Zhou et al. 2020). In contrast to the *TcS* promoter, which contains large insertions or deletions associated with increased promoter activity and theacrine accumulation in RY tea (Zhong et al. 2022), the *CsTcS2* promoter does not exhibit major structural variation (Figure S5). This implies that differential *CsTcS2* activation among cultivars is more likely driven by trans‐factor recruitment to conserved cis‐elements than by promoter rearrangement.

Among predicted regulators, CsTINY, an AP2/ERF family member in the DREB‐A4 subgroup, was prioritised based on high inferred binding affinity (score 13.72, Figure 3b), structural interaction confidence from AlphaFold 3 modelling (Figure S8a,b; Data S4) and co‐expression patterns (Figure 3d, Data S2). DREB‐type AP2/ERF TFs are known to recognise dehydration‐responsive elements (DREs) and to connect abiotic signals, developmental programs and specialised metabolic pathways (Machens et al. 2014; Sun et al. 2008; Wilson et al. 1996). CsTINY binds a DRE‐like motif (GTCGGC) in the *CsTcS2* promoter and activates promoter activity in DLR assays (7.86‐fold activation; Figure 3d; Figure 3f–h). Silencing *CsTINY* reduced both *CsTcS2* transcript abundance and theacrine accumulation (Figure 3e), indicating that CsTINY functions as an upstream activator of *CsTcS2*.

Taken together, these observations support a regulatory model in which CsTINY functions as a direct transcriptional activator of *CsTcS2* by binding a DRE‐like motif in its promoter and enhancing its transcriptional output. In other plant systems, DREB/ERF factors modulate specialised metabolite pathways either by activating biosynthetic genes directly or by modifying the activity of other transcriptional regulators. Examples include ERF factors that modulate anthocyanin accumulation in lily by interfering with the MYB‐bHLH‐WD40 complex (Yang et al. 2025), VqERF062 that controls stilbene biosynthesis in grapevine (Yan et al. 2025) and ERF189/ERF221 that regulate alkaloid biosynthesis in *Nicotiana* spp. and *Catharanthus roseus* (Feng et al. 2020; van der Fits and Memelink 2001). Therefore, CsTINY extends this regulatory logic to purine alkaloid metabolism in tea by acting as a direct transcriptional activator of *CsTcS2*, thereby linking an AP2/ERF TF to theacrine biosynthesis.

### 3.3 CsWRKY33 Regulates *CsTcS2* Expression to Elevate Theacrine Biosynthesis

WRKY TFs are widely recognised as integrators of defence‐related signalling and regulators of specialised metabolic pathways via direct promoter binding at W‐box or W‐box‐like motifs (Luo et al. 2022; Ren et al. 2023; Teng et al. 2021). CsWRKY33 was identified here as a second upstream activator of *CsTcS2*. AlphaFold 3 structural modelling revealed close spatial proximity between CsWRKY33, CsTINY and the *CsTcS2* promoter (Figure 4f), consistent with coordinated promoter occupancy. EMSA indicated that CsWRKY33 recognised a promoter motif (AACTTTT) in *CsTcS2* (Figure 5e) that resembled the WT‐box motif bound by certain WRKY factors such as AtWRKY50 and AtWRKY70, rather than the canonical TGAC core of the W‐box (Hussain et al. 2018; Machens et al. 2014). This suggests that *CsTcS2* promoter architecture and CsWRKY33 DNA‐binding specificity have co‐adapted. Such co‐adaptation of non‐canonical WRKY binding sites has been observed in multiple species in association with specialised metabolite pathway control (Li et al. 2020; Liu et al. 2023; Zhang, Wang, et al. 2024), including jasmonate‐responsive and defence‐related metabolites (Li et al. 2022; Zhang, Lu, et al. 2024 ).

In addition, CsWRKY33 behaves as a positive transcriptional regulator of *CsTcS2* (Figure 6). It activates the *CsTcS2* promoter in DLR assay (Figure 5c) and *CsWRKY33* silencing decreases *CsTcS2* expression and reduces theacrine content (Figure 7g). This places CsWRKY33 functionally in line with previously described WRKY regulators of specialised metabolic pathways, such as CjWRKY1 in berberine biosynthesis in *Coptis japonica* (Kato et al. 2007). NnWRKY40a/NnWRKY40b and NnWRKY70a/NnWRKY70b in benzylisoquinoline alkaloid regulation in lotus via jasmonate signalling (Li et al. 2022; Zhang, Lu, et al. 2024), and CsWRKY70 in purple‐leaf tea, which activates volatile biosynthetic genes such as *CsLOX3* and *CsTPS13* (Gao et al. 2025). Thus, the regulatory architecture of purine alkaloid metabolism in tea is consistent with broader principles observed in other specialised metabolic pathways in plants, in which WRKY TFs directly modulate branch‐point biosynthetic enzymes and thereby influence metabolic flux.

### 3.4 CsWRKY33‐CsTINY Module Synergistically Regulates *CsTcS2* Expression to Enhance Theacrine Accumulation

The combined activity of CsTINY and CsWRKY33 at the *CsTcS2* promoter defines a cooperative transcriptional module with coincidence‐detection properties, in which robust activation of *CsTcS2* occurs when both TFs are present and engaged at the promoter. Several independent lines of evidence support this model. Functionally, CsWRKY33 and CsTINY act cooperatively at the promoter level. Y2H, BiFC Co‐IP, LCA analyses indicate that CsWRKY33 and CsTINY physically interact and co‐localise at the promoter in planta (Figure 4a–e, Data S6). Co‐expression of both TFs results in higher *CsTcS2* promoter activity than either factor alone in DLR assays (Figure 7f), and transient overexpression in tea leaves increases *CsTcS2* transcript abundance and elevates theacrine accumulation (Figure 7a–c). By contrast, silencing *CsWRKY33* reduces the transcript levels of both *CsTINY* and *CsTcS2* and decreases theacrine content (Figure 7g), indicating regulatory interdependence between these two TFs in controlling *CsTcS2* expression and, consequently, metabolic flux through the theacrine branch of purine alkaloid metabolism.

Cooperative regulation of specialised metabolism by AP2/ERF and WRKY has been documented in other plant systems. WRKY41 and ERF jointly modulate anthocyanin biosynthesis (Sun et al. 2023), and in *Salvia miltiorrhiza* , SmWRKY1 and SmERF115 co‐activate phenolic acid biosynthesis (Cao et al. 2018). In persimmon, DkERF24 and DkWRKY1 interact and activate the *DkPDC2* promoter, facilitating responses to high‐CO_2_ or hypoxia in persimmon fruit and in *Arabidopsis* (Zhu et al. 2019). In wheat, TaRAP2‐13 L and TaWRKY10 co‐expression regulates abscisic acid biosynthetic genes and ROS‐scavenging genes, thereby enhancing drought tolerance (Shao et al. 2025). In soybean, GmERF1 and GmWRKY6 cooperate to confer low‐phosphate tolerance (Wang, Liu, Wei, et al. 2023 ). Consistent with these paradigms, the suggests that an analogous AP2/ERF‐WRKY cooperative module operates in purine alkaloid metabolism in tea, in which the CsWRKY33‐CsTINY module coordinates *CsTcS2* promoter activation to sustain elevated theacrine accumulation in high‐theacrine cultivars (Figure 8).

Although this study advances our understanding of the regulatory mechanism underlying theacrine biosynthesis, several questions remain. It is still unclear whether additional regulators (e.g., other TFs, co‐regulators, or non‐coding RNAs) modulate *CsTcS2* expression, and which upstream developmental or environmental signals drive CsTINY and CsWRKY33. In addition, the spatiotemporal regulation of *CsTcS2*, CsTINY and CsWRKY33 across tissues and stages should be resolved, as such dynamics likely contribute to tissue‐specific theacrine accumulation and may inform harvest‐related strategies.

## 4 Methods

### 4.1 Plant Materials

Tea samples were collected from different cultivars, including ‘Kucha3’ (‘KC3’), ‘Kucha2’ (‘KC2’), ‘Xianghong3’ (‘XH’) and ‘Shuchazao’ (‘SCZ’). For each cultivar, we sampled an apical bud with two young leaves (one bud and two leaves). In addition, for ‘KC3’, multiple tissues were collected, including buds, the first to fourth leaves, stems, roots and fruits. These samples were collected from the tea plantation of the Hunan Academy of Agricultural Sciences in Changsha, Hunan Province, China. Healthy tissues from each cultivar were obtained, and the fresh tea samples were immediately frozen in liquid nitrogen and stored at −80°C for subsequent use.

### 4.2 High‐Performance Liquid Chromatography (HPLC) Analysis

HPLC analysis was conducted using a Shimadzu LC‐20 ad‐PDA system, with an ECOSIL C18 column (4.6 × 150 mm, 5 μm, C/N EC181546, S/N 4I7501‐11). The column temperature was maintained at 35°C. The flow rate and injection volume were set to 1 mL·min^−1^ and 10 μL, respectively. The mobile phase consisted of (A) ultra‐pure water and (B) a mixture of 92% N,N‐dimethylformamide, 5% methanol and 3% acetic acid. The separation was performed under the following gradient conditions: 0–5 min: 9%–14% B; 5–8 min: 14% to 23% B; 8–10 min: 23% to 36% B; 10–12 min: 36% to 9% B; equilibration at 9% B until 15 min. Standard compounds, including 1,3,7‐trimethyluric acid, caffeine and theacrine (purity ≥ 98%), were used for compound identification and quantification.

### 4.3 Qualitative Analysis Using Ultra‐High‐Performance Liquid Chromatography–Triple Quadrupole Mass Spectrometry (UHPLC‐QqQ‐MS)

The qualitative analysis by UHPLC‐QqQ‐MS was adapted from the method described by Li, Xu, et al. (2024). The ultra‐high‐performance liquid chromatography system was coupled with a triple quadrupole mass spectrometer. All samples were separated on an ACQUITY UPLC BEH Amide column (2.1 × 100 mm, 1.7 μm) maintained at 35°C. The binary mobile phase was delivered at a flow rate of 0.3 mL/min, where solvent A consisted of 0.8% (v/v) acetic acid and 10 mM ammonium acetate in water, and solvent B contained acetonitrile with 0.1% (v/v) acetic acid. The gradient elution program was as follows: 0–2 min, 98% B; 2–3 min, 90% B; 3–6 min, 60% B; 6–7.5 min, 70% B; 7.5–8 min, 98% B; 8–10 min, 98% B. Mass spectrometry (MS) was conducted using electrospray ionisation (ESI) in both positive and negative ion modes. The ion source temperature was set to 450°C, and the ionisation voltage was −4500 V. Collision gas was used at medium pressure, with curtain gas (CUR) set at 10 psi, atomised gas at 40 psi and auxiliary gas at 60 psi. Multi‐reaction monitoring (MRM) mode was employed for detection.

### 4.4 Molecular Docking

Protein and DNA structural models were predicted using AlphaFold 3 (Abramson et al. 2024), with key target structures obtained from the Protein Data Bank (PDB). The compounds were converted to 3D structures using Open Babel (O'Boyle et al. 2011). Protein optimisation, including the removal of water molecules, was performed using PyMOL 2.1.0 (Eberhardt et al. 2021; Trott and Olson 2010). For protein–protein docking, HEX 8.0 software was used for global blind docking (Macindoe et al. 2010), employing a dense sampling strategy and clustering solutions based on directional similarity. The docking calculations incorporated shape complementarity and optional vacuum electrostatic contributions, with a rotation increment of 5.625° for the molecular axis and a 20‐sided polyhedron with 812 vertices for the remaining degrees of freedom. The binding affinity (kcal/mol) values represent the strength of the protein–protein interaction, with lower values indicating more stable binding (Dash et al. 2021; Tuo et al. 2024).

### 4.5 Transient Expression in Tea Plant

Branches of ‘KC3’ seedlings displaying similar growth trends were taken and cultured in water for a duration of 2 days. The fourth leaf was selected for transient overexpression. *A. tumefaciens* GV3101‐psoup‐p19 harbouring 35S::CsTcS2‐EYFP or 35S::EYFP vectors were injected into the leaf, after 48 h of culture, the injected part was taken for detection. Following Zhang, Wang, et al. (2024) and Jiao et al. (2023) described method.

### 4.6 Silencing of Gene in Tea Plant Leaves

Gene silencing experiments were conducted according to the method described by Wang et al. (2024), using AsODN to inhibit the expression of *CsTcS2*, *CsTINY* and *CsWRKY33* genes in the tender shoots of tea plants. AsODN were designed and selected using the Soligo software (Ding and Lawrence 2003), plants infiltrated with gene‐specific AsODN. Untreated plants grown under the same conditions and immersed in sODN served as the control. After 24 h of treatment (Hong et al. 2023), leaf samples were collected for HPLC analysis to measure changes in compound content. Additionally, qRT‐PCR was used to assess the changes in gene expression.

### 4.7 Gene Expression Analysis

Total RNA was extracted from tea leaves using the SteadyPure Plant Total RNA Isolation Kit. The RNA was reverse transcribed into cDNA using the FastKing One‐step RT‐PCR Kit, with 1000 ng of total RNA as the template. The resulting cDNA was used as a template for qRT‐PCR analysis and candidate gene cloning. Gene expression levels in tea plants were normalised to the GAPDH gene (TEA003029.1). The primers used for gene expression analysis are listed in Data S3.

### 4.8 Transcription Factor Prediction

The 2‐kb upstream promoter sequence of *CsTcS2* was extracted from the tea reference genome using TBtools. Tea TF motif profiles were obtained and annotated using pyJASPAR, and putative TF binding sites within the *CsTcS2* promoter were scanned using TFBSTools in R. Predicted binding was evaluated by the relative profile score, and matches with scores ≥ 95% were retained as high‐confidence sites. Candidate TFs were further prioritised based on co‐expression with *CsTcS2* by calculating Pearson correlation coefficients in R across integrated tea transcriptome datasets (tpia.teaplant.org; Chen et al. 2022; Jin et al. 2024a). The intersection of the top 20 TFs by motif‐match score and the top 20 TFs by expression correlation was retained for experimental validation.

### 4.9 Subcellular Localisation

The coding sequences of *CsTINY* and *CsWRKY33*, excluding the stop codons, were cloned into the pSuper1300‐GFP vector (Jin et al. 2024b), resulting in plant expression vectors for GFP fusion proteins. *Agrobacterium* strains carrying the constructs were infiltrated into tobacco leaves together with nuclear and membrane localisation signal markers. GFP signals from the recombinant proteins were detected using a laser scanning confocal microscope.

### 4.10 Yeast One‐Hybrid (Y1H) Assay

The promoter region of *CsTcS2* (−700 to −1100 bp) was cloned into the pHis2 vector. The CsTINY and CsWRKY33 were ligated to the yeast expression vector pGADT7, generating recombinant plasmids pGADT7‐CsTINY and pGADT7‐CsWRKY33. Yeast strain Y187 was co‐transformed with these plasmids and incubated for 2–3 days. Yeast cells were then diluted and plated on SD/‐His/−Leu/−Trp medium containing various concentrations of 3‐amino‐1,2,4‐triazole (3‐AT) for 2–3 days, with the AD vector serving as the negative control.

### 4.11 Electrophoretic Mobility Shift Assay (EMSA)

The CDS of *CsTINY* and the core region of *CsWRKY33* (631–1368 bp) were cloned into the pMAL‐MBP vector, which contains a recombinant maltose‐binding protein (MBP) tag. The constructs were transformed into *Escherichia coli* Rosetta (DE3) pLys competent cells, and protein expression was induced with isopropyl‐β‐D‐thiogalactopyranoside (IPTG). After 16 h of incubation at 16°C, CsTINY‐MBP and CsWRKY33‐MBP proteins were purified from the bacterial cells for further use in EMSA.

The EMSA experiment was performed with modifications based on the method of Jin et al. (2024b). A fragment of the *CsTcS2* promoter containing the corresponding transcription factor binding site was selected to synthesise a universal probe for protein‐DNA binding analysis. The competitive probe had the same sequence as the universal probe. For the mutant probe, the core binding site sequence was replaced with a single adenine (A), while the remainder of the sequence remained identical to the universal probe. All probes were used at a final concentration of 0.5 μM in the EMSA reactions.

### 4.12 Dual‐Luciferase Reporter (DLR) Assay

The DLR was performed following the method described by Hong et al. (2023). The *CsTcS2* promoter region was cloned into the pGreenII 0800‐LUC vector as the reporter gene. The full‐length genes of *CsTINY* and *CsWRKY33* were cloned into the pGreenII 62‐SK vector to generate effector constructs, which were then transformed into *Agrobacterium* strain GV3101 ‐psoup‐p19. The effector constructs and the reporter gene were co‐infiltrated into 4 week old tobacco leaves, using different combinations of effectors. After 1 day of dark incubation and 1 day of light incubation, leaf samples were collected for in vivo fluorescence imaging. The luciferase activity was measured using the TransDetect Double‐Luciferase Reporter Assay Kit, and the luminescence values of LUC and REN were determined to calculate their ratio.

### 4.13 Yeast Two‐Hybrid (Y2H) Assay

The coding sequence of *CsTINY* was cloned into the pGBKT7 vector to create the bait protein. The recombinant plasmid was transformed into *Saccharomyces cerevisiae* strain AH109. The *CsWRKY33* gene was cloned into the pGADT7 vector to produce the prey protein. Both the bait and prey proteins were co‐transformed into the AH109 yeast strain, and the transformants were plated on SD‐Trp/Leu plates and incubated for 2–3 days. Positive clones were selected and streaked onto SD‐Trp/−Leu and SD‐Ade‐His‐Leu‐Trp plates containing various concentrations of 3‐AT. The plates were incubated at 30°C for 3 days, and colony growth was monitored to confirm interactions.

### 4.14 Bimolecular Fluorescence Complementation (BiFC) Assay

The BiFC assay was performed as described by Xie et al. (2019). The coding sequence of *CsTINY* (without the stop codon) was cloned into the N‐terminal YFP fragment of the BiFC vector, while *CsWRKY33* was cloned into the C‐terminal YFP fragment. The recombinant plasmids were transferred into *Agrobacterium* strain GV3101. The two plasmids were co‐infiltrated into tobacco leaves for 2 days to allow expression and stable transmission of the recombinant DNA into plant cells. Fluorescence signals from the YFP‐tagged proteins were detected using a laser scanning confocal microscope.

### 4.15 Luciferase Complementation (LCA) Assay

The LCA was conducted following the protocol described by Sun et al. (2024). The coding sequence of *CsWRKY33* (without the stop codon) was cloned into the C‐terminal region of the LUC gene, and *CsTINY* was cloned into the N‐terminal region of the LUC gene (using pCAMBIA1300‐CLuc and pCAMBIA1300‐NLuc vectors). The recombinant plasmids were transformed into *Agrobacterium* strains, and the strains were used to inject 4–6 week old tobacco leaves. Leaf tissue expressing the experimental proteins was collected, and luciferase activity was measured using a live plant imaging system to detect bioluminescence intensity.

### 4.16 Co‐Immunoprecipitation (Co‐IP) Assay

For the Co‐IP assay, the CsTINY‐EYFP fusion construct was generated by inserting *CsTINY* into the pCAMBIA1300‐35S‐EYFP vector. To express HA‐tagged CsWRKY33, a CsWRKY33‐HA construct was generated using a vector retaining the native stop codon to ensure proper termination. The CsTINY‐EYFP and CsWRKY33‐HA constructs were co‐expressed in *N. benthamiana* leaves via Agrobacterium‐mediated infiltration. After 2 days of infiltration, infiltrated tobacco leaves were harvested and total proteins were extracted using Plant IP Lysis Buffer. Approximately 0.3 g of fresh or flash‐frozen leaf tissue was ground in liquid nitrogen using a pre‐chilled, sterile mortar and pestle. The homogenised tissue was washed twice with ice‐cold PBS, and excess PBS was removed before lysis. Protein concentration was determined using the BCA Protein Assay Kit. For input controls, an aliquot of the total protein extract was directly subjected to western blot (WB) analysis to confirm expression of the target proteins. The remaining lysate was pre‐cleared with 80 μL of protein A/G agarose beads at 4°C for 10 min to remove non‐specific proteins. The mixture was then centrifuged at 14000 × g for 15 min at 4°C, and the supernatant was collected. For immunoprecipitation, 1.0 μg of mouse IgG (negative control) or 1.0 μg of specific primary antibody (anti‐HA) was added to the respective lysate samples and incubated overnight at 4°C with gentle rotation. Subsequently, 20 μL of protein A/G beads was added to each sample and incubated for 2 h at room temperature. After centrifugation at 3000 rpm for 5 min at 4°C, the supernatant was discarded, and the beads were washed 3 times with cold PBS. The bound protein complexes were eluted and analysed by immunoblotting using anti‐EGFP antibody and anti‐HA antibody.

### 4.17 Statistical Analyses

Data are presented as mean ± standard deviation with error bars. For pairwise group comparisons, inter‐group significance was assessed through two‐tailed *t* tests implemented in GraphPad Prism 10. When analysing datasets containing three or more experimental groups, we employed one‐way ANOVA combined with Duncan's multiple range test using IBM SPSS software (version 19.0).

## Author Contributions

Z.L., J.H., N.T. and S.L. conceived the study, participated in the coordination, reviewed the manuscript and finalised the manuscript. T.W. designed and performed the experiments and drafted the paper. L.Z. analysed the data and prepared the figures and tables. C.S., S.X., N.L., X.L., F.W., L.P., H.J. and S.F. participated in the experiments and data analysis All authors read and approved the final manuscript.

## Funding

This work was supported by the National Key Research and Development Program of China, No. 2021YFD1200203. The National Natural Science Foundation of China, U22A20500, 32494780, 32172629. Hunan Agriculture Research System, HARS–10.

## Conflicts of Interest

The authors declare no conflicts of interest.

## Supporting information


**Data S1:** CkTcS sequence alignment in different genomes.

**Data S2:** Predicted candidate transcription factors of *CsTcS2*.

**Data S3:** Primer information used in this study.

**Data S4:** Predicted one‐to‐one binding of CsTINY protein to the *CsTcS2* promoter.

**Data S5:** Predicted one‐to‐one interaction between CsTINY and CsWRKY33 proteins.

**Data S6:** Yeast library construction.

**Data S7:** SNP and indel data of the promoter and gene region of *CsTcS2*.

**Figure S1:** Chromosome localisation of *CsTcS2*. The gene *CsTcS2* is located on chromosome Chr01 (GWHASIV00000001) from position 43 757 717 to 43 775 343 on the negative strand.
**Figure S2:** LC–MS‐based qualitative analysis of extracts. (a) Extract ion chromatogram of standard substances. Blue indicates 1,3,7‐trimethyluric acid (TMU), green represents theacrine (TC) and red represents caffeine (CAF). (b) Identification of 1,3,7‐trimethyluric acid in the Sample by spectrum at *m/z* 209.3. (c) Identification of theacrine in the Sample by spectrum at *m/z* 225.1. (d) Identification of caffeine in the Sample by spectrum at *m/z* 195.1.
**Figure S3:** Enzymatic characterisation of CsTcS2 with 1,3,7‐trimethyluric acid as substrate. (a) Kinetic comparison between CkTcS and CsTcS2 using 1,3,7‐trimethyluric acid. (b) SDS‐PAGE analysis of MBP‐CsTcS2 purification.
**Figure S4:** *CsTcS2* sequence analysis. (a) Domain prediction performed by InterProScan showed that CsTcS2 belongs to the Methyltransf_7 superfamily (PFAM: PF03492). (b) Alignment of the genomic sequence with the highest similarity against *CkTcS* and *CsTcS2*. (c) Coding sequence alignment of *CsTcS2* across four cultivars (‘Kucha3’, ‘Kucha2’, ‘Xianghong3’ and ‘Shuchazao’).
**Figure S5:** Heatmap of *CsTcS2* promoter variation. *CsTcS2* promoter molecular variants showed no differences, with no large‐scale insertions or deletions observed, based on 129 resequencing data sets. *CsTcS2* promoter.
**Figure S6:** Correlation analysis between the transcription factors and *CsTcS2*. On the basis of qPCR expression data from bitter tea and conventional tea cultivars, Pearson's correlation coefficients (r) were calculated between transcription factors and *CsTcS2*. Values in the upper triangle indicate Pearson's r, whereas the lower triangle shows pie charts representing the strength and direction of correlations. The colour scale ranges from −1 to 1, with purple indicating positive correlations and green indicating negative correlations; darker colours denote stronger correlations.
**Figure S7:** Pearson correlation analysis of *CsTcS2*, *CsTINY* and *CsWRKY33* expression levels with theacrine content across different tissues of ‘KC3’. The upper triangle shows Pearson correlation coefficients, while the lower triangle displays pie charts representing correlation strength and direction (purple indicates positive correlation). *CsTcS2* exhibits a strong positive correlation with theacrine content, and *CsTINY* shows the highest consistency with both *CsTcS*2 expression and theacrine accumulation. *CsWRKY33* displays a moderate but consistent positive correlation, suggesting its role as a cooperative regulator contributing to tissue‐specific activation of the theacrine biosynthetic pathway.
**Figure S8:** Two‐dimensional visualisation analysis of sequence depth search and transcription factor binding prediction in gene promoter region. (a) Structural schematic of CsTINY binding to the *CsTcS2* promoter region. The double‐helix represents promoter DNA, and the schematic above indicates the secondary structure of CsTINY. (b) Two‐dimensional visualisation analysis of the binding of a CsTINY and *CsTcS2* promoters. (c) Two‐dimensional visualisation analysis of the binding of CsTINY and CsWRKY33 to the *CsTcS2* promoter. The figure shows the results of sequence depth (coverage) analysis and prediction of potential DNA‐protein binding hotspots in the gene promoter region. The horizontal axis represents the base position (Positions) in the genome, and the vertical axis represents the corresponding sequence (Sequences). The colour gradient in the figure (from red to green) illustrates the spatial distribution of binding likelihood or binding strength. The darker the colour (red), the higher the predicted binding confidence. The black contour lines at the edges describe the coverage boundaries of gene regions. The colour scale on the right (from 0 to 1) corresponds to the numerical values of binding likelihood or binding probability. Areas in red have scores close to 0, while areas in green have scores close to 1, indicating potential protein‐DNA binding sites in specific regions. The peaks of CsTINY appeared at 0–200 and CsWRKY33 appeared at 600–800, representing two obvious binding hot spots, which may represent the key regulatory elements of specific binding between CsTINY and CsWRKY33.
**Figure S9:** SDS‐PAGE analysis confirming purification of MBP‐CsTINY fusion protein.
**Figure S10:** Yeast double hybrid screening. To identify proteins interacting with CsTINY, we conducted a yeast two‐hybrid (Y2H) screen using CsTINY as bait against a tea ( *Camellia sinensis* ) cDNA library. (a) Primary yeast library capacity identification. (b). Nuclear system secondary library capacity identification. (c). Membrane system secondary library capacity identification. (d). The yeast double hybrid screening library CsTINY was used as the bait protein.
**Figure S11:** Comparison of *CsCDH* expression and 1,3,7‐trimethyluric acid content between the tea cultivars ‘KC2’ and ‘XH’ (***p < 0.001, two‐tailed 11 Student's *t* test).
**Figure S12:** Semi‐quantitative RT‐PCR analysis of *CsTcS2* expression using cDNA from theacrine‐producing and non‐theacrine‐producing tea accessions as templates.

## Data Availability

All data, models and code generated or used during the study appear in the submitted article.

## References

[pbi70665-bib-0001] Abramson, J. , J. Adler , J. Dunger , et al. 2024. “Accurate Structure Prediction of Biomolecular Interactions With AlphaFold 3.” Nature 630: 493–500. 38718835 10.1038/s41586-024-07487-w PMC11168924

[pbi70665-bib-0002] Ashihara, H. , H. Sano , and A. Crozier . 2008. “Caffeine and Related Purine Alkaloids: Biosynthesis, Catabolism, Function and Genetic Engineering.” Phytochemistry 69: 841–856. 18068204 10.1016/j.phytochem.2007.10.029

[pbi70665-bib-0003] Bouillé, A. , R. Larbat , R. Kumari , et al. 2025. “Lineage‐Specific Patterns in the Moraceae Family Allow Identification of Convergent P450 Enzymes Involved in Furanocoumarin Biosynthesis.” New Phytologist 245: 2085–2102. 39776411 10.1111/nph.20381

[pbi70665-bib-0004] Cao, W. , Y. Wang , M. Shi , et al. 2018. “Transcription Factor SmWRKY1 Positively Promotes the Biosynthesis of Tanshinones in *Salvia miltiorrhiza* .” Frontiers in Plant Science 9: 554. 29755494 10.3389/fpls.2018.00554 PMC5934499

[pbi70665-bib-0005] Chen, C. , F. Xie , Q. Hua , et al. 2020. “Integrated sRNAome and RNA‐Seq Analysis Reveals miRNA Effects on Betalain Biosynthesis in Pitaya.” BMC Plant Biology 20: 437. 32962650 10.1186/s12870-020-02622-x PMC7510087

[pbi70665-bib-0006] Chen, L. , N. Tian , M. Hu , et al. 2022. “Comparative Transcriptome Analysis Reveals Key Pathways and Genes Involved in Trichome Development in Tea Plant ( *Camellia sinensis* ).” Frontiers in Plant Science 13: 997778. 36212317 10.3389/fpls.2022.997778 PMC9546587

[pbi70665-bib-0007] Chen, X. , S. Shao , R. Yang , et al. 2021. “Identification of Co‐Expressed Genes Related to Theacrine Synthesis in Tea Flowers at Different Developmental Stages.” International Journal of Molecular Sciences 22: 13394. 34948193 10.3390/ijms222413394 PMC8704887

[pbi70665-bib-0008] Cheng, L. , G. Tu , H. Ma , et al. 2024. “Alternative Splicing of CsbHLH133 Regulates Geraniol Biosynthesis in Tea Plants.” Plant Journal 120: 598–614. 10.1111/tpj.17003 39207906

[pbi70665-bib-0009] Dash, S. G. , S. Kantevari , S. K. Guru , and P. K. Naik . 2021. “Combination of Docetaxel and Newly Synthesized 9‐Br‐Trimethoxybenzyl‐Noscapine Improve Tubulin Binding and Enhances Antitumor Activity in Breast Cancer Cells.” Computers in Biology and Medicine 139: 104996. 34753081 10.1016/j.compbiomed.2021.104996

[pbi70665-bib-0010] Ding, Y. , and C. E. Lawrence . 2003. “A Statistical Sampling Algorithm for RNA Secondary Structure Prediction.” Nucleic Acids Research 31: 7280–7301. 14654704 10.1093/nar/gkg938 PMC297010

[pbi70665-bib-0011] Duan, W.‐J. , L. Liang , M.‐H. Pan , et al. 2020. “Theacrine, a Purine Alkaloid From Kucha, Protects Against Parkinson's Disease Through SIRT3 Activation.” Phytomedicine 77: 153281. 32707370 10.1016/j.phymed.2020.153281

[pbi70665-bib-0012] Eberhardt, J. , D. Santos‐Martins , A. F. Tillack , and S. Forli . 2021. “AutoDock Vina 1.2.0: New Docking Methods, Expanded Force Field, and Python Bindings.” Journal of Chemical Information and Modeling 61: 3891–3898. 34278794 10.1021/acs.jcim.1c00203 PMC10683950

[pbi70665-bib-0013] Feng, K. , X.‐L. Hou , G.‐M. Xing , et al. 2020. “Advances in AP2/ERF Super‐Family Transcription Factors in Plant.” Critical Reviews in Biotechnology 40: 750–776. 32522044 10.1080/07388551.2020.1768509

[pbi70665-bib-0014] Gao, C. , Z. Wang , Z. Chen , et al. 2025. “Integrated Volatile Metabolomics and Transcriptomics Analyses Revealed the Regulatory Mechanism of Aroma Compound Synthesis Induced by Microenvironmental Changes in Purple Tea Leaves.” Plant, Cell & Environment 48: 7168–7185. 10.1111/pce.70007 40525455

[pbi70665-bib-0015] Hayashi, S. , M. Watanabe , M. Kobayashi , T. Tohge , T. Hashimoto , and T. Shoji . 2020. “Genetic Manipulation of Transcriptional Regulators Alters Nicotine Biosynthesis in Tobacco.” Plant and Cell Physiology 61: 1041–1053. 32191315 10.1093/pcp/pcaa036

[pbi70665-bib-0016] Hong, G. , X. Zhang , L. Li , et al. 2023. “The CsHSFA‐CsJAZ6 Module‐Mediated High Temperature Regulates Flavonoid Metabolism in *Camellia sinensis* .” Plant, Cell & Environment 46: 2401–2418. 10.1111/pce.14610 37190917

[pbi70665-bib-0017] Hussain, R. M. F. , A. H. Sheikh , I. Haider , M. Quareshy , and H. J. M. Linthorst . 2018. “Arabidopsis WRKY50 and TGA Transcription Factors Synergistically Activate Expression of PR1.” Frontiers in Plant Science 9: 930. 30057584 10.3389/fpls.2018.00930 PMC6053526

[pbi70665-bib-0018] Jia, X. , S. Luo , X. Ye , L. Liu , and W. Wen . 2024. “Evolution of the Biochemistry Underpinning Purine Alkaloid Metabolism in Plants.” Philosophical Transactions of the Royal Society, B: Biological Sciences 379: 20230366. 10.1098/rstb.2023.0366 PMC11449220 39343019

[pbi70665-bib-0019] Jiang, D. , L. Xu , and W. Wen . 2025. “A Novel Transcription Factor CsSNACA2 Plays a Pivotal Role Within Nitrogen Assimilation in Tea Plants.” Plant Journal 121: e17198. 10.1111/tpj.17198 39661731

[pbi70665-bib-0020] Jiao, T. , Y. Huang , Y.‐L. Wu , et al. 2023. “Functional Diversity of Subgroup 5 R2R3‐MYBs Promoting Proanthocyanidin Biosynthesis and Their Key Residues and Motifs in Tea Plant.” Horticulture Research 10: uhad135. 37694228 10.1093/hr/uhad135 PMC10484168

[pbi70665-bib-0021] Jin, J. , Y. Xu , P. Lu , et al. 2020. “Degradome, Small RNAs and Transcriptome Sequencing of a High‐Nicotine Cultivated Tobacco Uncovers Mirna's Function in Nicotine Biosynthesis.” Scientific Reports 10: 11751. 32678207 10.1038/s41598-020-68691-y PMC7366715

[pbi70665-bib-0022] Jin, J.‐Q. , Y.‐F. Chai , Y.‐F. Liu , J. Zhang , M.‐Z. Yao , and L. Chen . 2018. “Hongyacha, a Naturally Caffeine‐Free Tea Plant From Fujian, China.” Journal of Agricultural and Food Chemistry 66: 11311–11319. 30303011 10.1021/acs.jafc.8b03433

[pbi70665-bib-0023] Jin, J.‐Q. , C. Zhou , C. Ma , M.‐Z. Yao , J. Ma , and L. Chen . 2014. “Identification on Purine Alkaloids of Representative Tea Germplasms in China.” Journal of Plant Genetic Resources 15: 279–285.

[pbi70665-bib-0024] Jin, Q. , Z. Wang , D. Sandhu , et al. 2024a. “mRNA‐miRNA Analyses Reveal the Involvement of CsbHLH1 and miR1446a in the Regulation of Caffeine Biosynthesis in *Camellia sinensis* .” Horticulture Research 11: uhad282. 39686959 10.1093/hr/uhad282 PMC11648165

[pbi70665-bib-0025] Jin, Q. , Z. Wang , D. Sandhu , et al. 2024b. “miR828a‐CsMYB114 Module Negatively Regulates the Biosynthesis of Theobromine in *Camellia sinensis* .” Journal of Agricultural and Food Chemistry 72: 4464–4475. 38376143 10.1021/acs.jafc.3c07736

[pbi70665-bib-0026] Juliano, L. M. , and R. R. Griffiths . 2004. “A Critical Review of Caffeine Withdrawal: Empirical Validation of Symptoms and Signs, Incidence, Severity, and Associated Features.” Psychopharmacology 176: 1–29. 15448977 10.1007/s00213-004-2000-x

[pbi70665-bib-0027] Kato, N. , E. Dubouzet , Y. Kokabu , et al. 2007. “Identification of a WRKY Protein as a Transcriptional Regulator of Benzylisoquinoline Alkaloid Biosynthesis in *Coptis japonica* .” Plant and Cell Physiology 48: 8–18. 17132631 10.1093/pcp/pcl041

[pbi70665-bib-0028] Li, C. , J. Wu , K.‐D. Hu , et al. 2020. “PyWRKY26 and PybHLH3 Cotargeted the PyMYB114 Promoter to Regulate Anthocyanin Biosynthesis and Transport in Red‐Skinned Pears.” Horticulture Research 7: 37. 32194973 10.1038/s41438-020-0254-z PMC7072072

[pbi70665-bib-0029] Li, H. , K. Fang , D. Qin , et al. 2021. “Comparative Transcriptome Analysis Reveals Putative Genes Responsible for High Theacrine Content in Kucha (*Camellia kucha* [Chang et Wang] Chang).” Tropical Plant Biology 14: 82–92.

[pbi70665-bib-0030] Li, H. , H. He , M. Yan , et al. 2024. “CsmiR396d Targeting of CsGS2 Plays an Important Role in Glutamine Metabolism of Tea Plant ( *Camellia sinensis* ).” Beverage Plant Research 4: e005.

[pbi70665-bib-0031] Li, J. , Y. Li , M. Dang , et al. 2022. “Jasmonate‐Responsive Transcription Factors NnWRKY70a and NnWRKY70b Positively Regulate Benzylisoquinoline Alkaloid Biosynthesis in Lotus ( *Nelumbo nucifera* ).” Frontiers in Plant Science 13: 862915. 35783938 10.3389/fpls.2022.862915 PMC9240598

[pbi70665-bib-0032] Li, N. , J. Xu , Y. Zhao , et al. 2024. “The Influence of Processing Methods on Polyphenol Profiling of Tea Leaves From the Same Large‐Leaf Cultivar ( *Camellia sinensis* var. *assamica* cv. Yunkang‐10): Nontargeted/Targeted Polyphenomics and Electronic Sensory Analysis.” Food Chemistry 460: 140515. 39067433 10.1016/j.foodchem.2024.140515

[pbi70665-bib-0033] Li, W.‐X. , Y.‐F. Li , Y.‐J. Zhai , W.‐M. Chen , H. Kurihara , and R.‐R. He . 2013. “Theacrine, a Purine Alkaloid Obtained From *Camellia assamica* Var. *Kucha*, Attenuates Restraint Stress‐Provoked Liver Damage in Mice.” Journal of Agricultural and Food Chemistry 61: 6328–6335. 23678853 10.1021/jf400982c

[pbi70665-bib-0034] Liu, F. , T. Dou , C. Hu , et al. 2023. “WRKY Transcription Factor MaWRKY49 Positively Regulates Pectate Lyase Genes During Fruit Ripening of *Musa acuminata* .” Plant Physiology and Biochemistry 194: 643–650. 36535104 10.1016/j.plaphy.2022.12.015

[pbi70665-bib-0035] Liu, Y. , D. Pang , and Y. Li . 2022. “The Screening and Identification of Key Transcription Factor Genes for Theacrine Metabolism.” Journal of Tea Science 42: 41–50.

[pbi70665-bib-0036] Luo, Y. , X.‐X. Huang , X.‐F. Song , et al. 2022. “Identification of a WRKY Transcriptional Activator From *Camellia sinensis* That Regulates Methylated EGCG Biosynthesis.” Horticulture Research 9: uhac024. 35184160 10.1093/hr/uhac024 PMC9071374

[pbi70665-bib-0037] Lv, X.‐T. , R.‐H. Wang , X.‐T. Liu , et al. 2022. “Theacrine Ameliorates Experimental Liver Fibrosis in Rats by Lowering Cholesterol Storage via Activation of the Sirtuin 3‐Farnesoid X Receptor Signaling Pathway.” Chemico‐Biological Interactions 364: 110051. 35872049 10.1016/j.cbi.2022.110051

[pbi70665-bib-0038] Machens, F. , M. Becker , F. Umrath , and R. Hehl . 2014. “Identification of a Novel Type of WRKY Transcription Factor Binding Site in Elicitor‐Responsive Cis‐Sequences From *Arabidopsis thaliana* .” Plant Molecular Biology 84: 371–385. 24104863 10.1007/s11103-013-0136-y

[pbi70665-bib-0039] Macindoe, G. , L. Mavridis , V. Venkatraman , M.‐D. Devignes , and D. Ritchie . 2010. “HexServer: An FFT‐Based Protein Docking Server Powered by Graphics Processors.” Nucleic Acids Research 38: W445–W449. 20444869 10.1093/nar/gkq311 PMC2896144

[pbi70665-bib-0040] O'Boyle, N. M. , M. Banck , C. A. James , C. Morley , T. Vandermeersch , and G. R. Hutchison . 2011. “Open Babel: An Open Chemical Toolbox.” Journal of Cheminformatics 3: 33. 21982300 10.1186/1758-2946-3-33 PMC3198950

[pbi70665-bib-0041] Ouyang, S.‐H. , Y.‐J. Zhai , Y.‐P. Wu , et al. 2021. “Theacrine, a Potent Antidepressant Purine Alkaloid From a Special Chinese Tea, Promotes Adult Hippocampal Neurogenesis in Stressed Mice.” Journal of Agricultural and Food Chemistry 69: 7016–7027. 34060828 10.1021/acs.jafc.1c01514

[pbi70665-bib-0042] Pani, A. , and R. K. Mahapatra . 2013. “Computational Identification of microRNAs and Their Targets in *Catharanthus roseus* Expressed Sequence Tags.” Genomics Data 1: 2–6. 26484050 10.1016/j.gdata.2013.06.001 PMC4608865

[pbi70665-bib-0043] Ren, L. , W. Wan , D. Yin , et al. 2023. “Genome‐Wide Analysis of WRKY Transcription Factor Genes in *Toona sinensis*: An Insight Into Evolutionary Characteristics and Terpene Synthesis.” Frontiers in Plant Science 13: 1063850. 36743538 10.3389/fpls.2022.1063850 PMC9895799

[pbi70665-bib-0044] Shao, C. , Z. Gao , M. Sun , L. Xiang , X. Chen , and J. Wang . 2025. “The Drought‐Responsive Wheat AP2/ERF Transcription Factor TaRAP2‐13L and Its Interacting Protein TaWRKY10 Enhance Drought Tolerance in Transgenic *Arabidopsis* and Wheat ( *Triticum aestivum* L.).” International Journal of Biological Macromolecules 309: 143008. 40239777 10.1016/j.ijbiomac.2025.143008

[pbi70665-bib-0045] Shen, E. M. , S. K. Singh , J. S. Ghosh , et al. 2017. “The miRNAome of *Catharanthus roseus* : Identification, Expression Analysis, and Potential Roles of microRNAs in Regulation of Terpenoid Indole Alkaloid Biosynthesis.” Scientific Reports 7: 43027. 28223695 10.1038/srep43027 PMC5320439

[pbi70665-bib-0046] Singh, S. K. , B. Patra , P. Paul , Y. Liu , S. Pattanaik , and L. Yuan . 2020. “Revisiting the ORCA Gene Cluster That Regulates Terpenoid Indole Alkaloid Biosynthesis in *Catharanthus roseus* .” Plant Science 293: 110408. 32081258 10.1016/j.plantsci.2020.110408

[pbi70665-bib-0047] Sun, H. , K. Hu , S. Wei , G. Yao , and H. Zhang . 2023. “ETHYLENE RESPONSE FACTORS 4.1/4.2 With an EAR Motif Repress Anthocyanin Biosynthesis in Red‐Skinned Pears.” Plant Physiology 192: 1892–1912. 36732887 10.1093/plphys/kiad068 PMC10315276

[pbi70665-bib-0048] Sun, Q. , Z. He , R. Wei , et al. 2024. “The Transcriptional Regulatory Module CsHB5‐CsbZIP44 Positively Regulates Abscisic Acid‐Mediated Carotenoid Biosynthesis in Citrus (*Citrus* Spp.).” Plant Biotechnology Journal 22: 722–737. 37915111 10.1111/pbi.14219 PMC10893943

[pbi70665-bib-0049] Sun, S. , J.‐P. Yu , F. Chen , et al. 2008. “TINY, a Dehydration‐Responsive Element (DRE)‐Binding Protein‐Like Transcription Factor Connecting the DRE‐ and Ethylene‐Responsive Element‐Mediated Signaling Pathways in *Arabidopsis* .” Journal of Biological Chemistry 283: 6261–6271. 18089556 10.1074/jbc.M706800200

[pbi70665-bib-0050] Suttipanta, N. , S. Pattanaik , M. Kulshrestha , B. Patra , S. K. Singh , and L. Yuan . 2011. “The Transcription Factor CrWRKY1 Positively Regulates the Terpenoid Indole Alkaloid Biosynthesis in *Catharanthus roseus* .” Plant Physiology 157: 2081–2093. 21988879 10.1104/pp.111.181834 PMC3327198

[pbi70665-bib-0051] Tariq, A. , M. Meng , X. Jiang , et al. 2024. “In‐Depth Exploration of the Genomic Diversity in Tea Varieties Based on a Newly Constructed Pangenome of *Camellia sinensis* .” Plant Journal 119: 2096–2115. 10.1111/tpj.16874 38872506

[pbi70665-bib-0052] Teng, J. , C. Yan , W. Zeng , Y. Zhang , Z. Zeng , and Y. Huang . 2020. “Purification and Characterization of Theobromine Synthase in a Theobromine‐Enriched Wild Tea Plant (*Camellia gymnogyna* Chang) From Dayao Mountain, China.” Food Chemistry 311: 125875. 31753680 10.1016/j.foodchem.2019.125875

[pbi70665-bib-0053] Teng, R. , Y. Wang , S. Lin , et al. 2021. “CsWRKY13, a Novel WRKY Transcription Factor of *Camellia sinensis* , Involved in Lignin Biosynthesis and Accumulation.” Beverage Plant Research 1: 12.

[pbi70665-bib-0054] Trott, O. , and A. J. Olson . 2010. “AutoDock Vina: Improving the Speed and Accuracy of Docking With a New Scoring Function, Efficient Optimization, and Multithreading.” Journal of Computational Chemistry 31: 455–461. 19499576 10.1002/jcc.21334 PMC3041641

[pbi70665-bib-0055] Tuo, Y. , X. Lu , F. Tao , et al. 2024. “The Potential Mechanisms of Catechins in Tea for Anti‐Hypertension: An Integration of Network Pharmacology, Molecular Docking, and Molecular Dynamics Simulation.” Food 13: 2685. 10.3390/foods13172685 PMC11394219 39272451

[pbi70665-bib-0056] van der Fits, L. , and J. Memelink . 2001. “The Jasmonate‐Inducible AP2/ERF‐Domain Transcription Factor ORCA3 Activates Gene Expression via Interaction With a Jasmonate‐Responsive Promoter Element.” Plant Journal 25: 43–53. 10.1046/j.1365-313x.2001.00932.x 11169181

[pbi70665-bib-0057] Wang, C. , X. Hao , Y. Wang , et al. 2022. “Identification of WRKY Transcription Factors Involved in Regulating the Biosynthesis of the Anti‐Cancer Drug Camptothecin in *Ophiorrhiza pumila* .” Horticulture Research 9: uhac099. 35795387 10.1093/hr/uhac099 PMC9250654

[pbi70665-bib-0058] Wang, J. , Y. Hu , D. Guo , et al. 2024. “Evolution and Functional Divergence of Glycosyltransferase Genes Shaped the Quality and Cold Tolerance of Tea Plants.” Plant Cell 37: koae268. 39365921 10.1093/plcell/koae268 PMC11663605

[pbi70665-bib-0059] Wang, R. , X. Liu , H. Zhu , et al. 2023. “Transcription Factors GmERF1 and GmWRKY6 Synergistically Regulate Low Phosphorus Tolerance in Soybean.” Plant Physiology 192: 1099–1114. 36932694 10.1093/plphys/kiad170 PMC10231356

[pbi70665-bib-0060] Wang, S. , J. Chen , J. Ma , J. Jin , L. Chen , and M. Yao . 2020. “Novel Insight Into Theacrine Metabolism Revealed by Transcriptome Analysis in Bitter Tea (Kucha, *Camellia sinensis* ).” Scientific Reports 10: 6286. 32286351 10.1038/s41598-020-62859-2 PMC7156766

[pbi70665-bib-0061] Wang, Y. , Y.‐F. Liu , M.‐Y. Wei , et al. 2023. “Deeply Functional Identification of TCS1 Alleles Provides Efficient Technical Paths for Low‐Caffeine Breeding of Tea Plants.” Horticulture Research 10: uhac279. 36793757 10.1093/hr/uhac279 PMC9926157

[pbi70665-bib-0062] Wang, Y. , X. Yang , X. Zheng , J. Li , C. Ye , and X. Song . 2010. “Theacrine, a Purine Alkaloid With Anti‐Inflammatory and Analgesic Activities.” Fitoterapia 81: 627–631. 20227468 10.1016/j.fitote.2010.03.008

[pbi70665-bib-0063] Wei, C. , H. Yang , S. Wang , et al. 2018. “Draft Genome Sequence of *Camellia sinensis* var. *sinensis* Provides Insights Into the Evolution of the Tea Genome and Tea Quality.” Proceedings of the National Academy of Sciences of the United States of America 115: E4151–E4158. 29678829 10.1073/pnas.1719622115 PMC5939082

[pbi70665-bib-0064] Wilson, K. , D. Long , J. Swinburne , and G. Coupland . 1996. “A Dissociation Insertion Causes a Semidominant Mutation That Increases Expression of TINY, an *Arabidopsis* Gene Related to APETALA2.” Plant Cell 8: 659–671. 8624440 10.1105/tpc.8.4.659 PMC161127

[pbi70665-bib-0065] Xie, Z. , T. Nolan , H. Jiang , et al. 2019. “The AP2/ERF Transcription Factor TINY Modulates Brassinosteroid‐Regulated Plant Growth and Drought Responses in *Arabidopsis* .” Plant Cell 31: 1788–1806. 31126980 10.1105/tpc.18.00918 PMC6713308

[pbi70665-bib-0066] Xu, J.‐K. , H. Kurihara , L. Zhao , and X.‐S. Yao . 2007. “Theacrine, a Special Purine Alkaloid With Sedative and Hypnotic Properties From *Camellia assamica* Var. *Kucha* in Mice.” Journal of Asian Natural Products Research 9: 665–672. 17943563 10.1080/10286020601103155

[pbi70665-bib-0067] Yamada, Y. , S. Nishida , N. Shitan , and F. Sato . 2020. “Genome‐Wide Identification of AP2/ERF Transcription Factor‐Encoding Genes in California Poppy ( *Eschscholzia californica* ) and Their Expression Profiles in Response to Methyl Jasmonate.” Scientific Reports 10: 18066. 33093564 10.1038/s41598-020-75069-7 PMC7582171

[pbi70665-bib-0068] Yan, C. , W. Liu , R. Li , G. Liu , and Y. Wang . 2025. “VqERF1B‐VqERF062‐VqNSTS2 Transcriptional Cascade Enhances Stilbene Biosynthesis and Resistance to Powdery Mildew in Grapevine.” Plant Biotechnology Journal 23: 2065–2082. 40062824 10.1111/pbi.70041 PMC12120875

[pbi70665-bib-0069] Yang, Y. , M. Bi , K. Luo , et al. 2025. “Lily (*Lilium* Spp.) LhERF061 Suppresses Anthocyanin Biosynthesis by Inhibiting LhMYBSPLATTER and LhDFR Expression and Interacting With LhMYBSPLATTER.” Plant Physiology and Biochemistry 219: 109325. 39612825 10.1016/j.plaphy.2024.109325

[pbi70665-bib-0070] Ye, Y. , H. Ashihara , X.‐Q. Zheng , X.‐J. Wang , K. Gao , and H.‐D. Zhang . 2003. “New Discovery of Pattern of Purine Alkaloids in Wild Tea Trees.” Journal of Sun Yat‐Sen University 42: 62–65.

[pbi70665-bib-0071] Ying, C. , J. Chen , J. Chen , et al. 2023. “Differential Accumulation Mechanisms of Purine Alkaloids and Catechins in *Camellia ptilophylla*, a Natural Theobromine‐Rich Tea.” Beverage Plant Research 3: 15.

[pbi70665-bib-0072] Zhang, F. , W. Li , C. Gao , D. Zhang , and L. Gao . 2019. “Deciphering Tea Tree Chloroplast and Mitochondrial Genomes of *Camellia sinensis* var. *assamica* .” Scientific Data 6: 209. 31624267 10.1038/s41597-019-0201-8 PMC6797725

[pbi70665-bib-0073] Zhang, F. , F. Qiu , J. Zeng , et al. 2023. “Revealing Evolution of Tropane Alkaloid Biosynthesis by Analyzing Two Genomes in the Solanaceae Family.” Nature Communications 14: 1446. 10.1038/s41467-023-37133-4 PMC10017790 36922496

[pbi70665-bib-0074] Zhang, M. , P. Lu , Y. Zheng , et al. 2024. “Genome‐Wide Identification of AP2/ERF Gene Family in *Coptis chinensis* Franch Reveals Its Role in Tissue‐Specific Accumulation of Benzylisoquinoline Alkaloids.” BMC Genomics 25: 972. 39415101 10.1186/s12864-024-10883-1 PMC11484470

[pbi70665-bib-0075] Zhang, S. , J. Jin , J. Chen , S. Ercisli , and L. Chen . 2022. “Purine Alkaloids in Tea Plants: Component, Biosynthetic Mechanism and Genetic Variation.” Beverage Plant Research 2: 13.

[pbi70665-bib-0076] Zhang, X. , S. Chen , L. Shi , et al. 2021. “Haplotype‐Resolved Genome Assembly Provides Insights Into Evolutionary History of the Tea Plant *Camellia sinensis* .” Nature Genetics 53: 1250–1259. 34267370 10.1038/s41588-021-00895-y PMC8346365

[pbi70665-bib-0077] Zhang, Y. , J. Fu , Q. Zhou , et al. 2022. “Metabolite Profiling and Transcriptome Analysis Revealed the Conserved Transcriptional Regulation Mechanism of Caffeine Biosynthesis in Tea and Coffee Plants.” Journal of Agricultural and Food Chemistry 70: 3239–3251. 35245048 10.1021/acs.jafc.1c06886

[pbi70665-bib-0078] Zhang, Y. , J. Wang , Y. Xiao , et al. 2024. “CsWRKY12 Interacts With CsVQ4L to Promote the Accumulation of Galloylated Catechins in Tender Leaves of Tea Plants.” Plant Journal 120: 2861–2873. 10.1111/tpj.17150 39570713

[pbi70665-bib-0079] Zhang, Y.‐H. , Y.‐F. Li , Y. Wang , et al. 2020. “Identification and Characterization of N9‐Methyltransferase Involved in Converting Caffeine Into Non‐Stimulatory Theacrine in Tea.” Nature Communications 11: 1473. 10.1038/s41467-020-15324-7 PMC7081346 32193380

[pbi70665-bib-0080] Zheng, X.‐Q. , C.‐X. Ye , M. Kato , A. Crozier , and H. Ashihara . 2002. “Theacrine (1,3,7,9‐Tetramethyluric Acid) Synthesis in Leaves of a Chinese Tea, Kucha (*Camellia assamica* Var. *Kucha*).” Phytochemistry 60: 129–134. 12009315 10.1016/s0031-9422(02)00086-9

[pbi70665-bib-0081] Zhong, H. , Y. Wang , F.‐R. Qu , et al. 2022. “A Novel TcS Allele Conferring the High‐Theacrine and Low‐Caffeine Traits and Having Potential Use in Tea Plant Breeding.” Horticulture Research 9: uhac191. 36338849 10.1093/hr/uhac191 PMC9630966

[pbi70665-bib-0082] Zhou, M. , and J. Memelink . 2016. “Jasmonate‐Responsive Transcription Factors Regulating Plant Secondary Metabolism.” Biotechnology Advances 34: 441–449. 26876016 10.1016/j.biotechadv.2016.02.004

[pbi70665-bib-0083] Zhou, M. , D. O'Neill Rothenberg , W. Zeng , et al. 2022. “Discovery and Biochemical Characterization of N‐Methyltransferase Genes Involved in Purine Alkaloid Biosynthetic Pathway of *Camellia gymnogyna* Hung T. Chang (Theaceae) From Dayao Mountain.” Phytochemistry 199: 113167. 35378107 10.1016/j.phytochem.2022.113167

[pbi70665-bib-0084] Zhou, M. , C. Yan , Z. Zeng , L. Luo , W. Zeng , and Y. Huang . 2020. “N‐Methyltransferases of Caffeine Biosynthetic Pathway in Plants.” Journal of Agricultural and Food Chemistry 68: 15359–15372. 33206517 10.1021/acs.jafc.0c06167

[pbi70665-bib-0085] Zhu, L. , T. Wu , C. Shao , et al. 2025. “Cloning and Functional Characterization of the Caffeine Oxidase Gene CsCDH From *Camellia sinensis* .” International Journal of Biological Macromolecules 302: 140429. 39884634 10.1016/j.ijbiomac.2025.140429

[pbi70665-bib-0086] Zhu, Q.‐G. , Z.‐Y. Gong , J. Huang , D. Grierson , K.‐S. Chen , and X.‐R. Yin . 2019. “High‐CO2/Hypoxia‐Responsive Transcription Factors DkERF24 and DkWRKY1 Interact and Activate DkPDC2 Promoter.” Plant Physiology 180: 621–633. 30850469 10.1104/pp.18.01552 PMC6501092

