# Supplementary figures and images for: Transcriptional Regulation of the Novel Theacrine Synthase Gene CsTcS2 by the CsTINY–CsWRKY33 Module Underpins Theacrine Biosynthesis in Camellia sinensis
Source: Plant Biotechnol J. 2026 Apr 15;24(8):4823–43. doi: 10.1111/pbi.70665 (PMC13387896; doi:10.1111/pbi.70665)

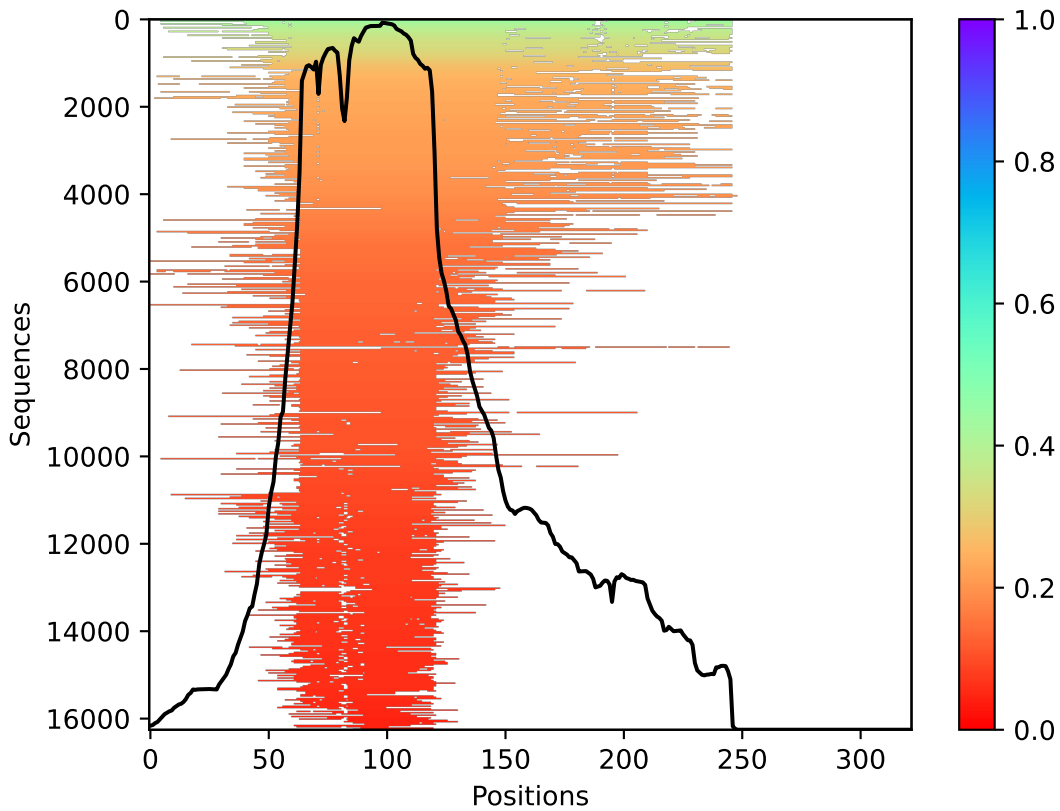

Supplement: Supplementary file 4 — Data S4: Predicted one‐to‐one binding of CsTINY protein to the CsTcS2 promoter. [file PBI-24-4823-s003.zip › msa_depth.pdf]

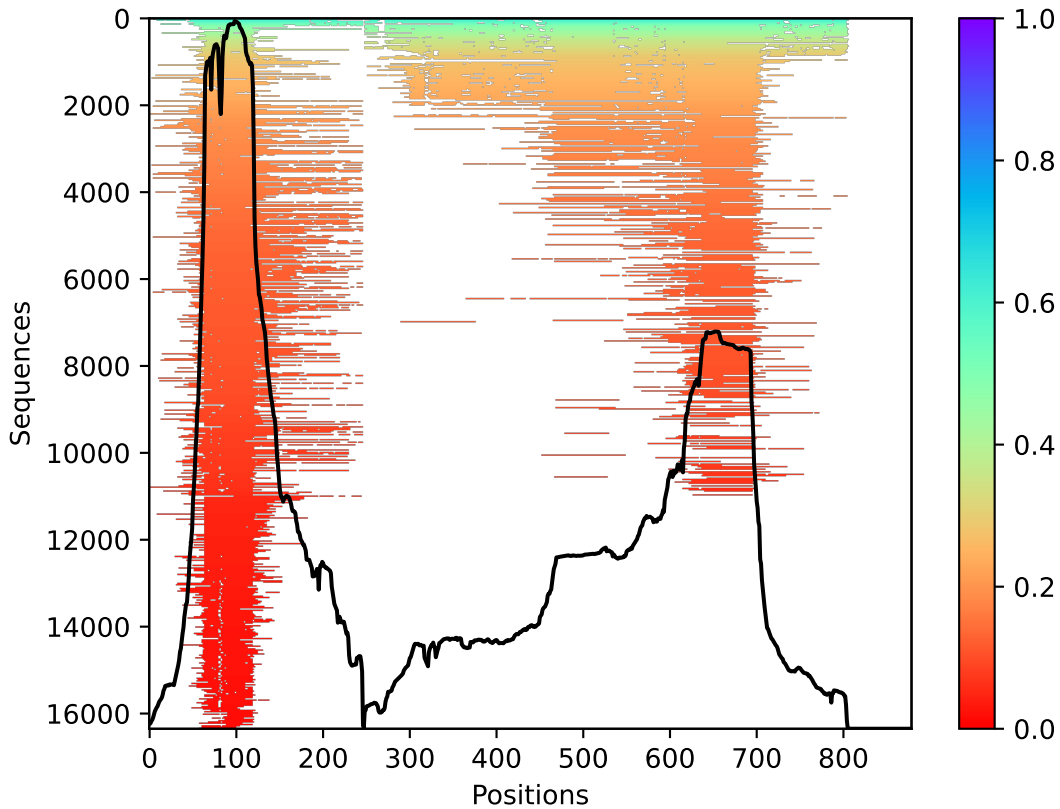

Supplement: Supplementary file 5 — Data S5: Predicted one‐to‐one interaction between CsTINY and CsWRKY33 proteins. [file PBI-24-4823-s005.zip › msa_depth.pdf]

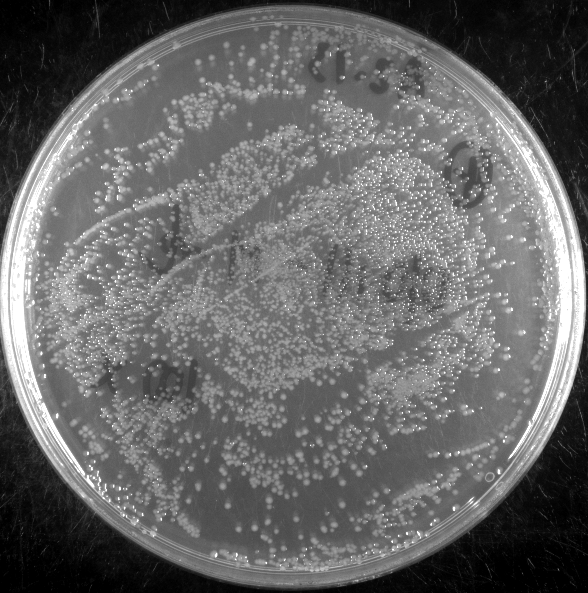

Supplement: Supplementary file 6 — Data S6: Yeast library construction. [file PBI-24-4823-s008.zip › Supplementary Data 6 Yeast library construction/6 . Identification of storage capacity of sub-catalogues in nuclear systems.bmp]

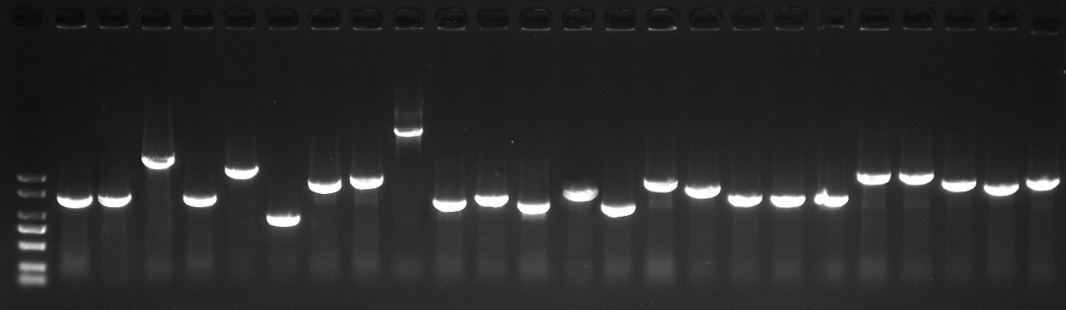

Supplement: Supplementary file 6 — Data S6: Yeast library construction. [file PBI-24-4823-s008.zip › Supplementary Data 6 Yeast library construction/5 . Primary library capacity appraisal.bmp]

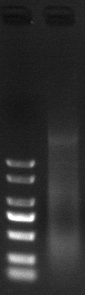

Supplement: Supplementary file 6 — Data S6: Yeast library construction. [file PBI-24-4823-s008.zip › Supplementary Data 6 Yeast library construction/1 . mRNA.bmp]

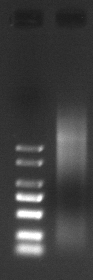

Supplement: Supplementary file 6 — Data S6: Yeast library construction. [file PBI-24-4823-s008.zip › Supplementary Data 6 Yeast library construction/2 . Two-strand DNA.bmp]

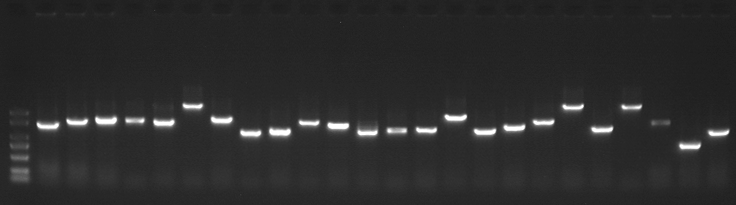

Supplement: Supplementary file 6 — Data S6: Yeast library construction. [file PBI-24-4823-s008.zip › Supplementary Data 6 Yeast library construction/3 . Cultures were identified in the primary library.bmp]

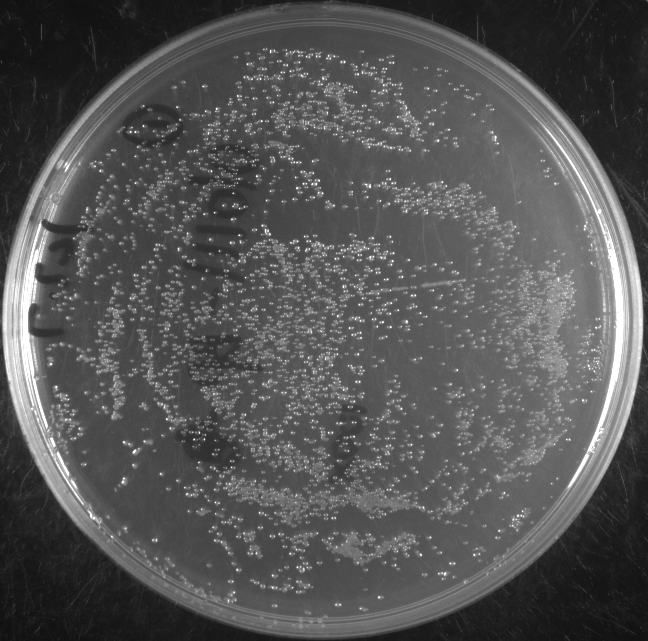

Supplement: Supplementary file 6 — Data S6: Yeast library construction. [file PBI-24-4823-s008.zip › Supplementary Data 6 Yeast library construction/4 . Primary library capacity appraisal.bmp]

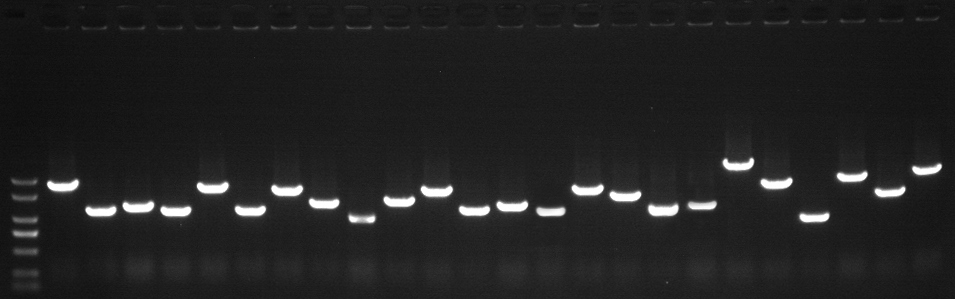

Supplement: Supplementary file 6 — Data S6: Yeast library construction. [file PBI-24-4823-s008.zip › Supplementary Data 6 Yeast library construction/7 . Secondary library colony identification of membrane system.bmp]

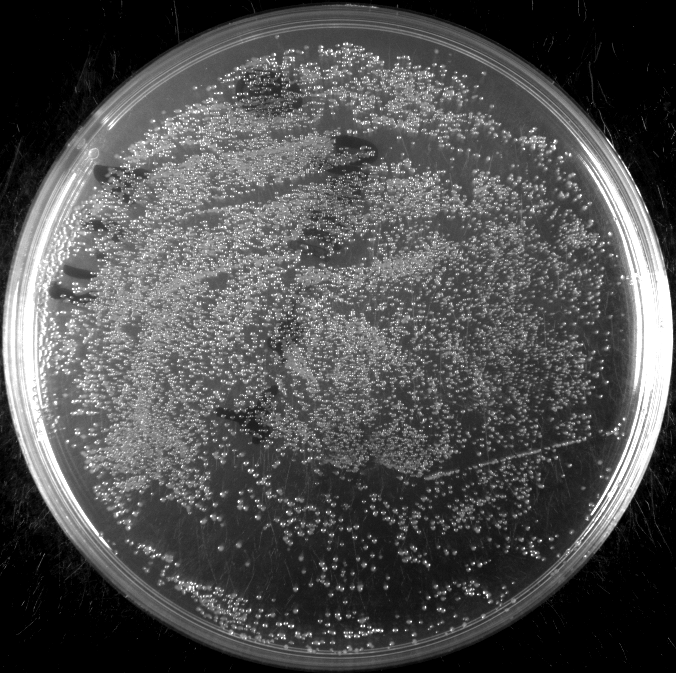

Supplement: Supplementary file 6 — Data S6: Yeast library construction. [file PBI-24-4823-s008.zip › Supplementary Data 6 Yeast library construction/8 . Identification of secondary library capacity of membrane system.bmp]
