# Supplementary material for: Transcriptional Regulation of the Novel Theacrine Synthase Gene CsTcS2 by the CsTINY–CsWRKY33 Module Underpins Theacrine Biosynthesis in Camellia sinensis
Source: Plant Biotechnol J. 2026 Apr 15;24(8):4823–43. doi: 10.1111/pbi.70665 (PMC13387896; doi:10.1111/pbi.70665)
Supplement: Supplementary file 8 — Figure S1: Chromosome localisation of CsTcS2. The gene CsTcS2 is located on chromosome Chr01 (GWHASIV00000001) from position 43 757 717 to 43 775 343 on the negative strand. Figure S2: LC–MS‐based qualitative analysis of extracts. (a) Extract ion chromatogram of standard substances. Blue indicates 1,3,7‐trimethyluric acid (TMU), green represents theacrine (TC) and red represents caffeine (CAF). (b) Identification of 1,3,7‐trimethyluric acid in the Sample by spectrum at m/z 209.3. (c) Identification of theacrine in the Sample by spectrum at m/z 225.1. (d) Identification of caffeine in the Sample by spectrum at m/z 195.1. Figure S3: Enzymatic characterisation of CsTcS2 with 1,3,7‐trimethyluric acid as substrate. (a) Kinetic comparison between CkTcS and CsTcS2 using 1,3,7‐trimethyluric acid. (b) SDS‐PAGE analysis of MBP‐CsTcS2 purification. Figure S4: CsTcS2 sequence analysis. (a) Domain prediction performed by InterProScan showed that CsTcS2 belongs to the Methyltransf_7 superfamily (PFAM: PF03492). (b) Alignment of the genomic sequence with the highest similarity against CkTcS and CsTcS2. (c) Coding sequence alignment of CsTcS2 across four cultivars (‘Kucha3’, ‘Kucha2’, ‘Xianghong3’ and ‘Shuchazao’). Figure S5: Heatmap of CsTcS2 promoter variation. CsTcS2 promoter molecular variants showed no differences, with no large‐scale insertions or deletions observed, based on 129 resequencing data sets. CsTcS2 promoter. Figure S6: Correlation analysis between the transcription factors and CsTcS2. On the basis of qPCR expression data from bitter tea and conventional tea cultivars, Pearson's correlation coefficients (r) were calculated between transcription factors and CsTcS2. Values in the upper triangle indicate Pearson's r, whereas the lower triangle shows pie charts representing the strength and direction of correlations. The colour scale ranges from −1 to 1, with purple indicating positive correlations and green indicating negative correlations; darker colours denote stron [file PBI-24-4823-s007.docx]

# **Supplemental Figures for Transcriptional regulation of the novel theacrine synthase gene CsTcS2 by the CsTINY-CsWRKY33 module underpins theacrine biosynthesis in Camellia** sinensis

Ting Wu^1,2,3,4,5^**^†^**, Lihua Zhu^1,3,5^**^†^**, Chenyu Shao^1,2,3^, Siyi Xie^1,2,4^, Na Li^1,3,4^, Xinyu Li^1,2,4^, Fang Wang^1,2,4^, Lvwen Peng^1,3,4^, Huiying Jin^1,3,4^, Fanghuizi Shang^1,2,4^, Jianan Huang^1,2,3^, Zhonghua Liu^1,2,3^*, Na Tian^1,3,4^*, Shuoqian Liu^1,2,3,4,5^*

^1^ State Key Laboratory of Tea Plant Germplasm Innovation and Resource Utilization, Hunan Agricultural University, Changsha, 410128, China

^2^ National Research Center of Engineering and Technology for Utilization of Botanical Functional Ingredients, Changsha, 410128, China

^3^ Yuelushan Laboratory, Changsha, 410128, China

^4^ Key Laboratory of Tea Science of Ministry of Education, Hunan Agricultural University, Changsha, 410128, China.

^5^ Key Laboratory for Evaluation and Utilization of Gene Resources of Horticultural Crops, Ministry of Agriculture and Rural Affairs of China, Hunan Agricultural University, Changsha, 410128, China

*** To whom correspondence should be addressed:**

Zhonghua Liu, Ph.D.

Professor

Email: [zhonghualiu163@163.com](mailto:zhonghualiu163@163.com)

Na Tian, Ph.D.

Professor

Email: [tianna5678@hunau.edu.cn](mailto:tianna5678@hunau.edu.cn,)

Shuoqian Liu, Ph.D.

Professor

Email: [shuoqianliu@hunau.edu.cn](mailto:shuoqianliu@hunau.edu.cn)

State Key Laboratory of Tea Plant Germplasm Innovation and Resource Utilization, Hunan Agricultural University, Changsha, 410128, China


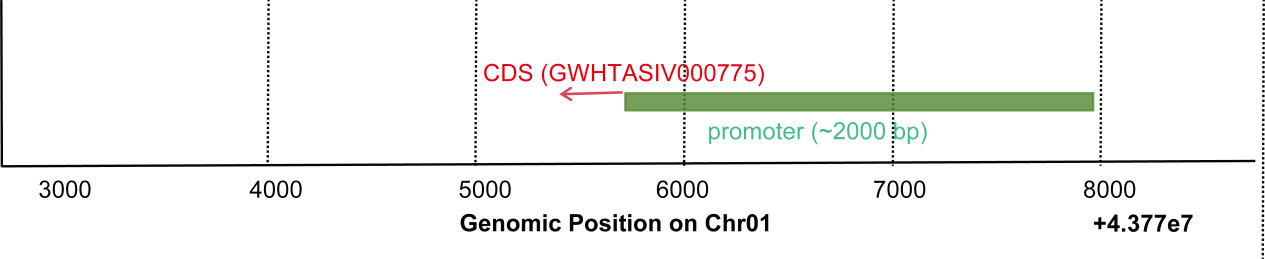


**Figure S1. Chromosome localization of *CsTcS2*.** The gene ***CsTcS2*** is located on chromosome Chr01 (GWHASIV00000001) from position 43,757,717 to 43,775,343 on the negative strand.


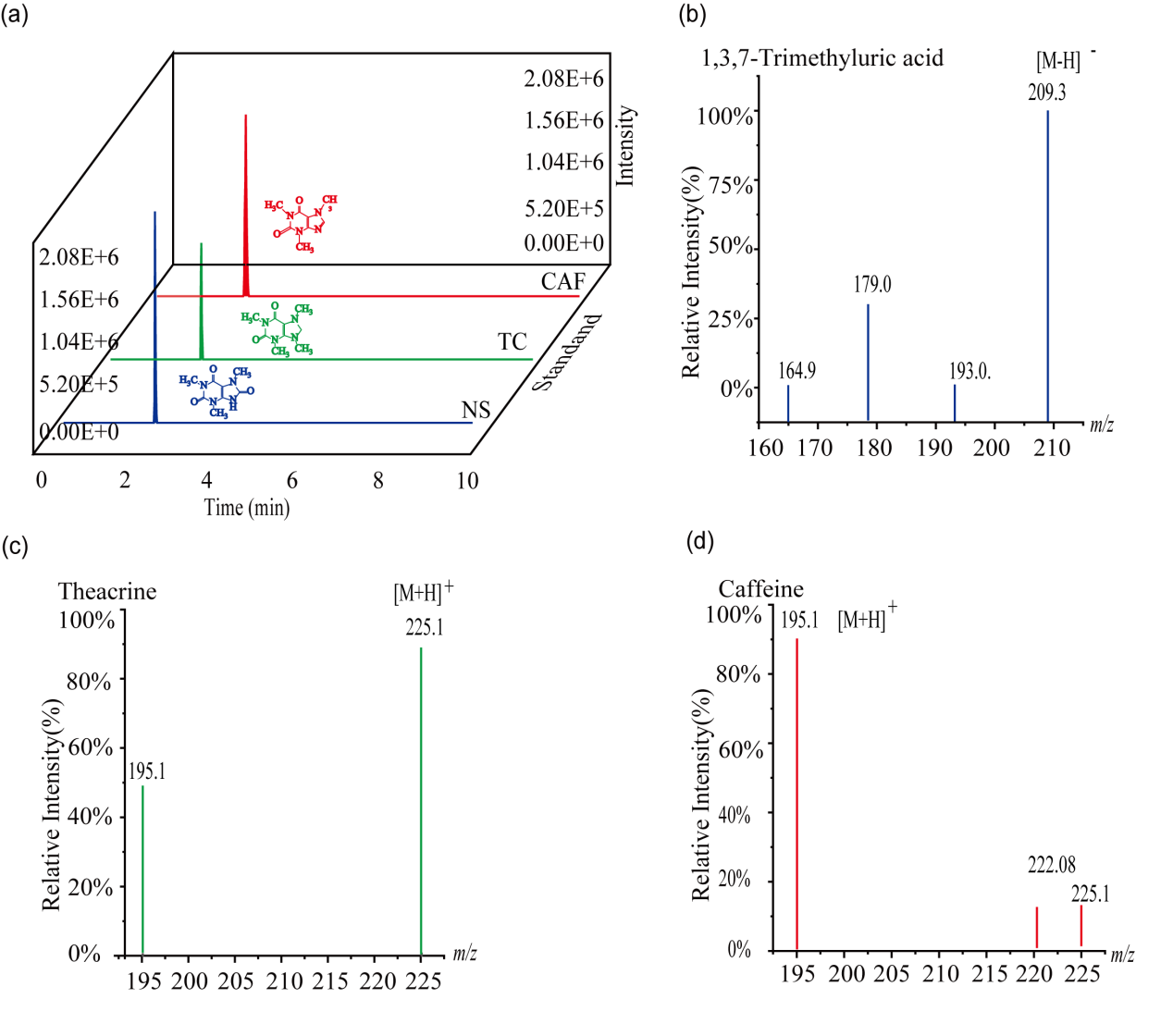


**Figure S2. LC-MS-based qualitative analysis of extracts.** (a) Extract ion chromatogram of standard substances. Blue indicates 1,3,7-trimethyluric acid (TMU), green represents theacrine (TC), and red represents caffeine (CAF). (b**)** Identification of 1,3,7-trimethyluric acid in the Sample by spectrum at *m/z* 209.3. (c) Identification of theacrine in the Sample by spectrum at *m/z* 225.1. (d) Identification of caffeine in the Sample by spectrum at *m/z* 195.1.


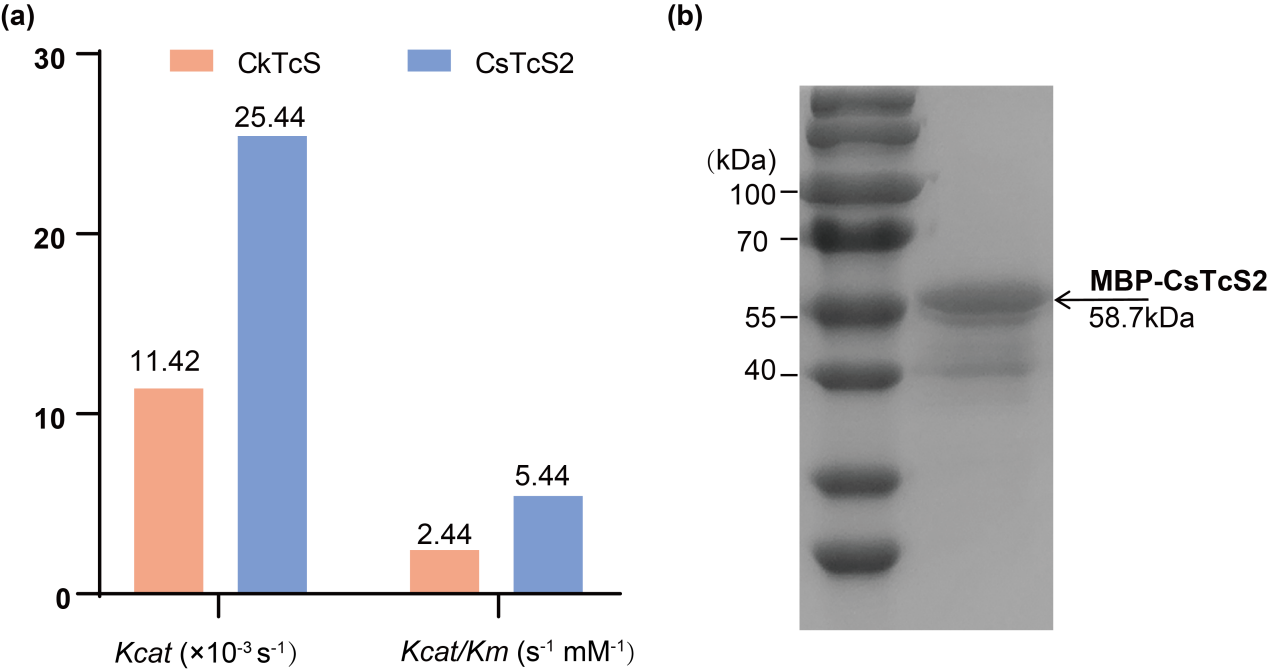
**Figure S3. Enzymatic characterization of CsTcS2 with 1,3,7-trimethyluric acid as substrate.** (a) Kinetic comparison between CkTcS and CsTcS2 using 1,3,7-trimethyluric acid. (b) SDS-PAGE analysis of MBP-CsTcS2 purification.


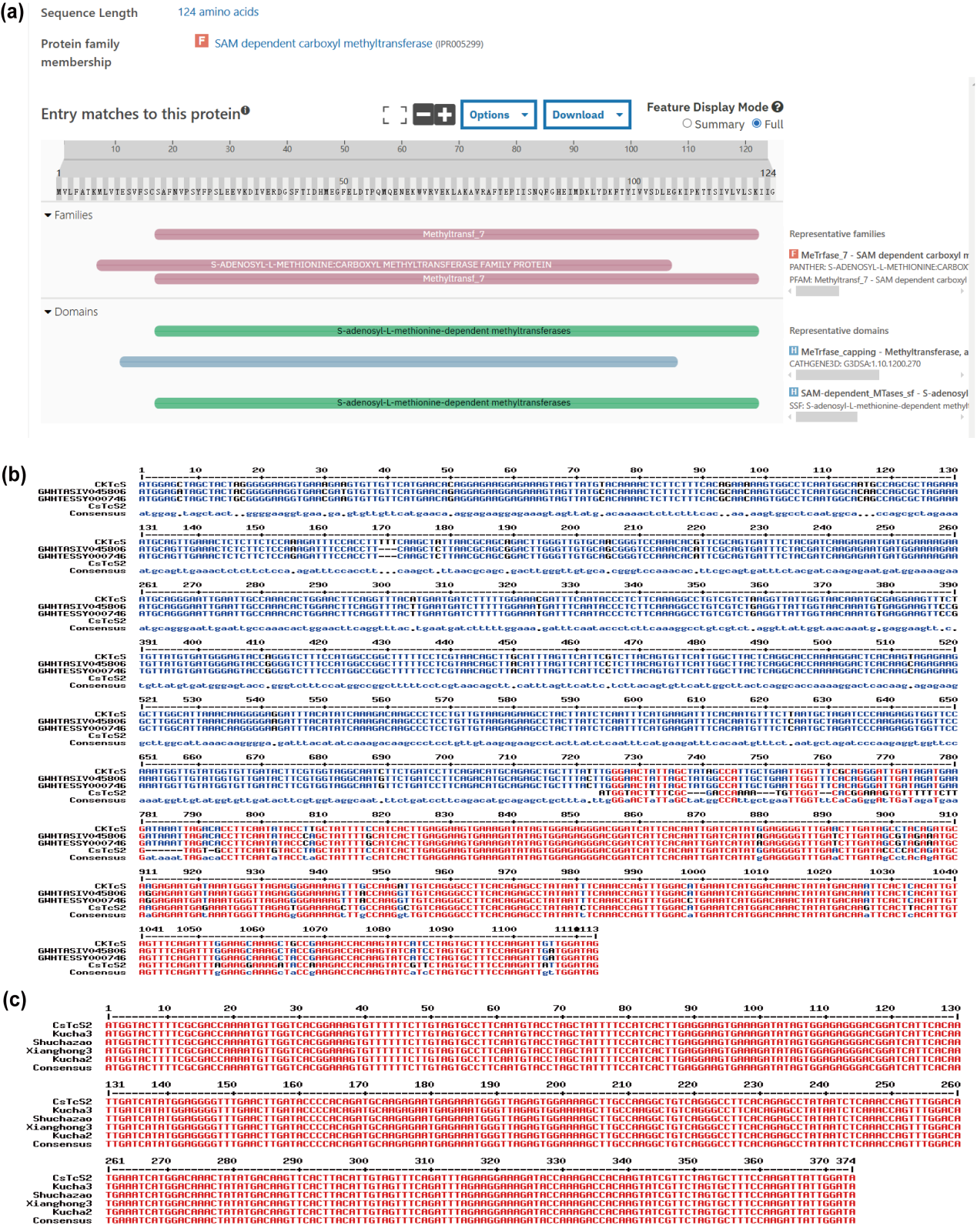


**Figure S4. *CsTcS2* sequence analysis.** (a) Domain prediction performed by InterProScan showed that CsTcS2 belongs to the Methyltransf_7 superfamily (PFAM: PF03492). (b) Alignment of the genomic sequence with the highest similarity against *CkTcS* and *CsTcS2*. (c) Coding sequence alignment of *CsTcS2* across four cultivars (‘Kucha3’, ‘Kucha2’, ‘Xianghong3’, and ‘Shuchazao’).


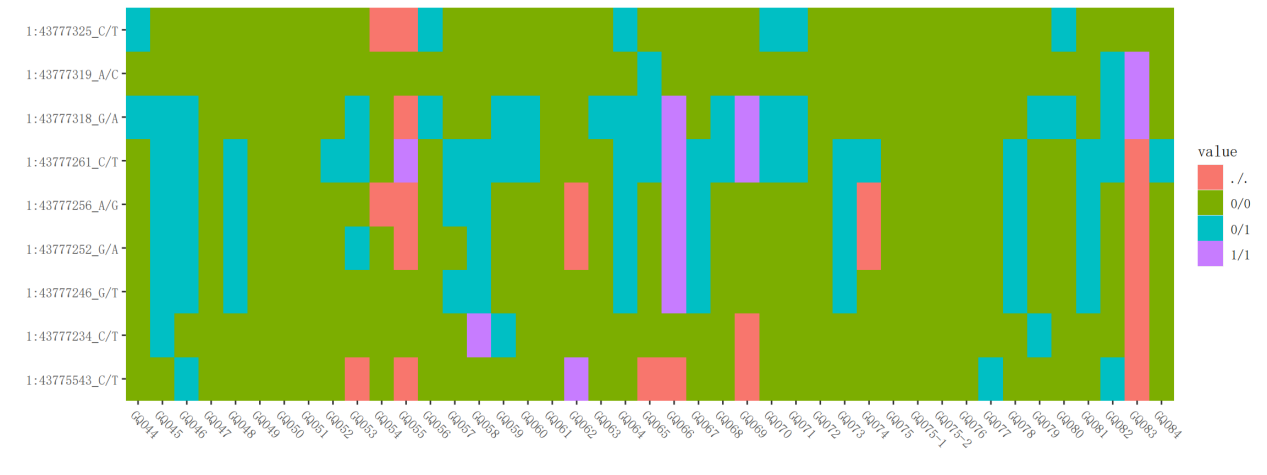

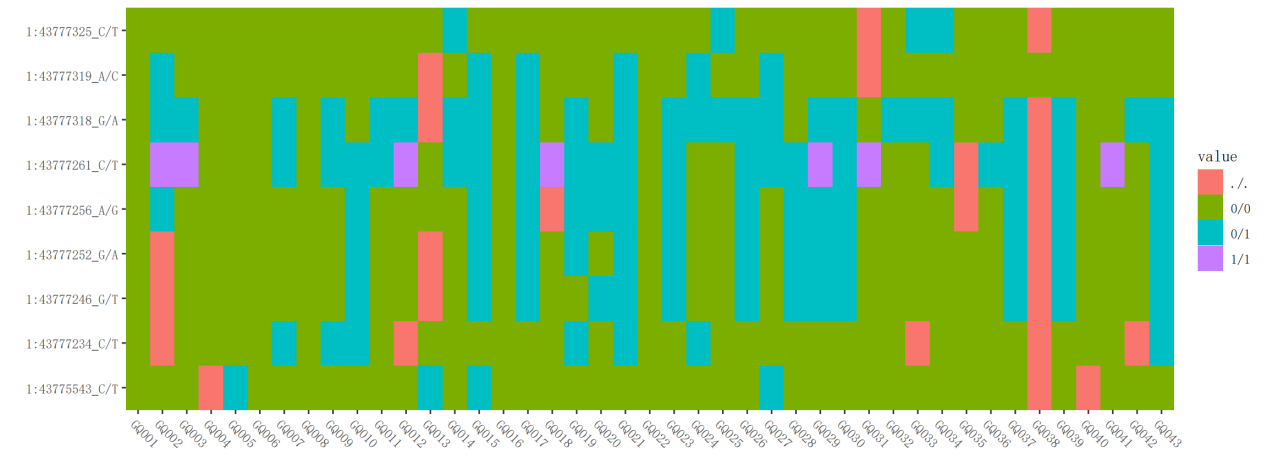

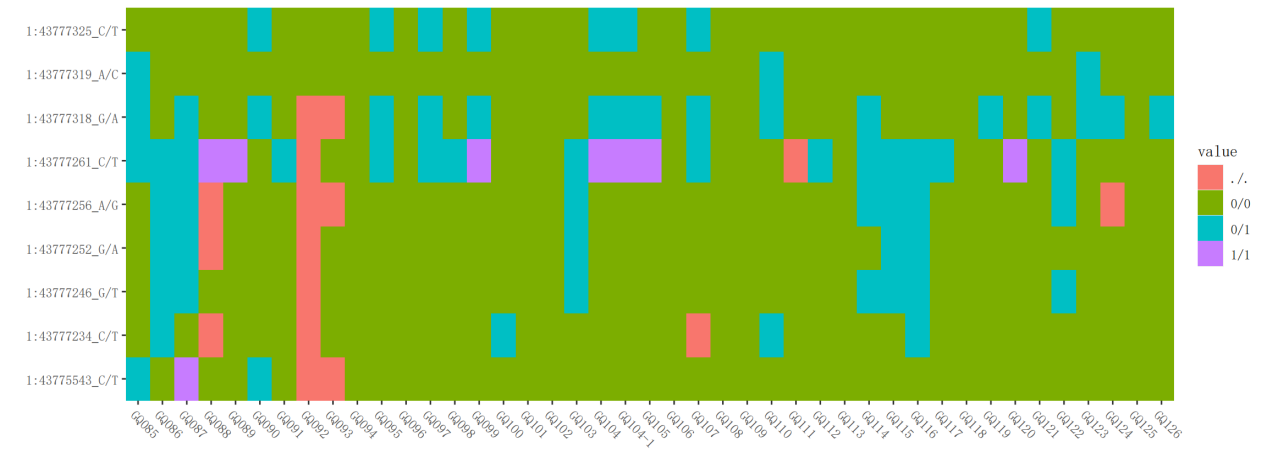


**Figure S5. Heatmap of *CsTcS2* promoter variation.** *CsTcS2* promoter molecular variants showed no differences, with no large-scale insertions or deletions observed, based on 129 resequencing data sets. *CsTcS2* promoter.

**
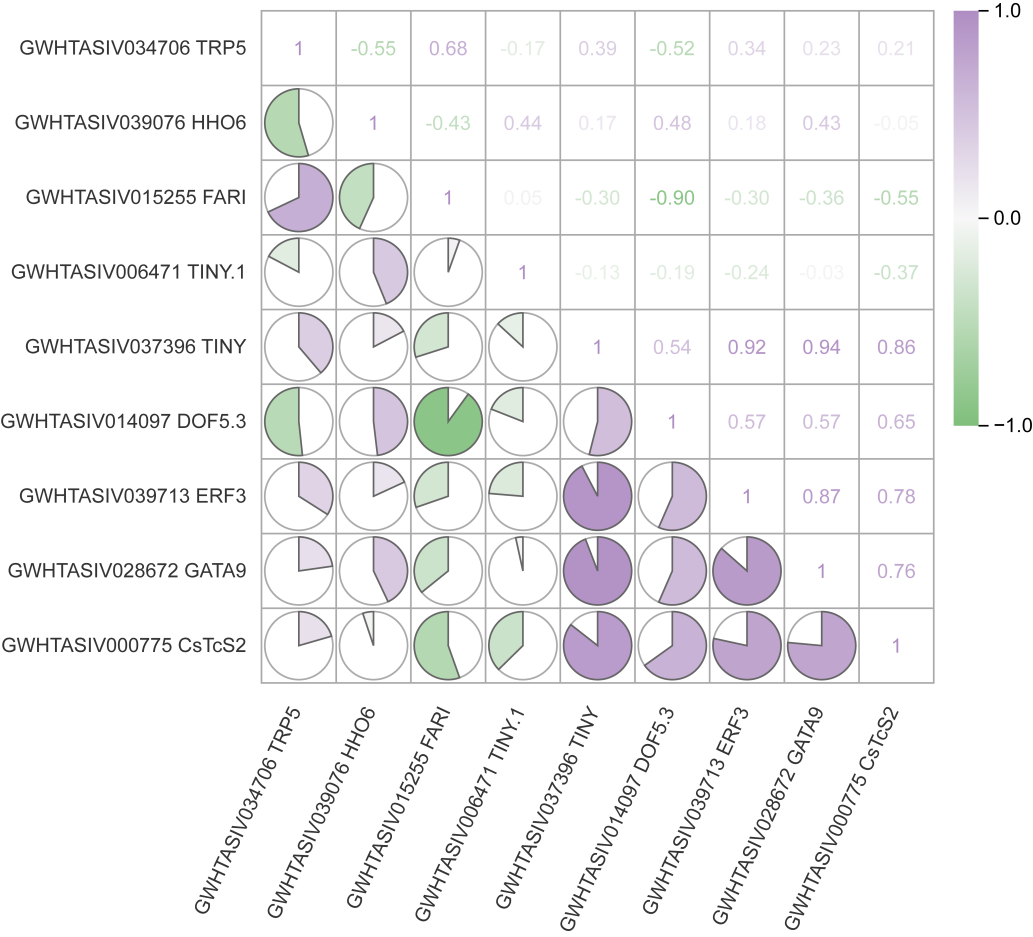
**

**Figure S6.** **Correlation analysis between the transcription factors and *CsTcS2*.** Based on qPCR expression data from bitter tea and conventional tea cultivars, Pearson’s correlation coefficients (r) were calculated between transcription factors and *CsTcS2*. Values in the upper triangle indicate Pearson’s r, whereas the lower triangle shows pie charts representing the strength and direction of correlations. The color scale ranges from −1 to 1, with purple indicating positive correlations and green indicating negative correlations; darker colors denote stronger correlations.


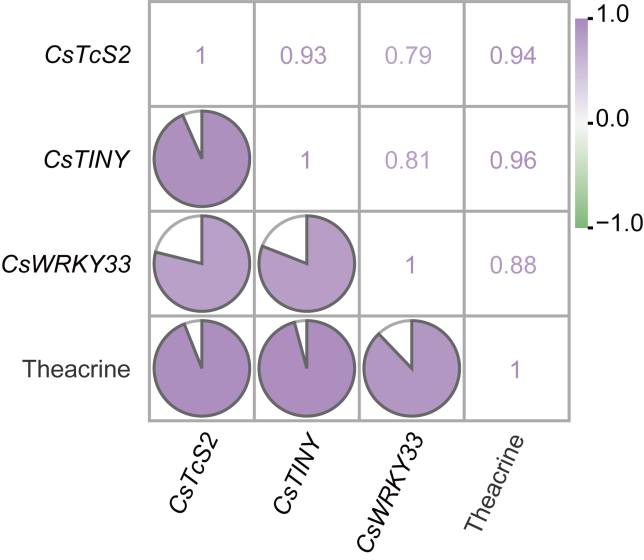


**Figure S7.** **Pearson correlation analysis of *CsTcS2*, *CsTINY*, and *CsWRKY33* expression levels with theacrine content across different tissues of ‘KC3’.** The upper triangle shows Pearson correlation coefficients, while the lower triangle displays pie charts representing correlation strength and direction (purple indicates positive correlation). *CsTcS2* exhibits a strong positive correlation with theacrine content, and *CsTINY* shows the highest consistency with both *CsTcS*2 expression and theacrine accumulation. *CsWRKY33* displays a moderate but consistent positive correlation, suggesting its role as a cooperative regulator contributing to tissue-specific activation of the theacrine biosynthetic pathway.


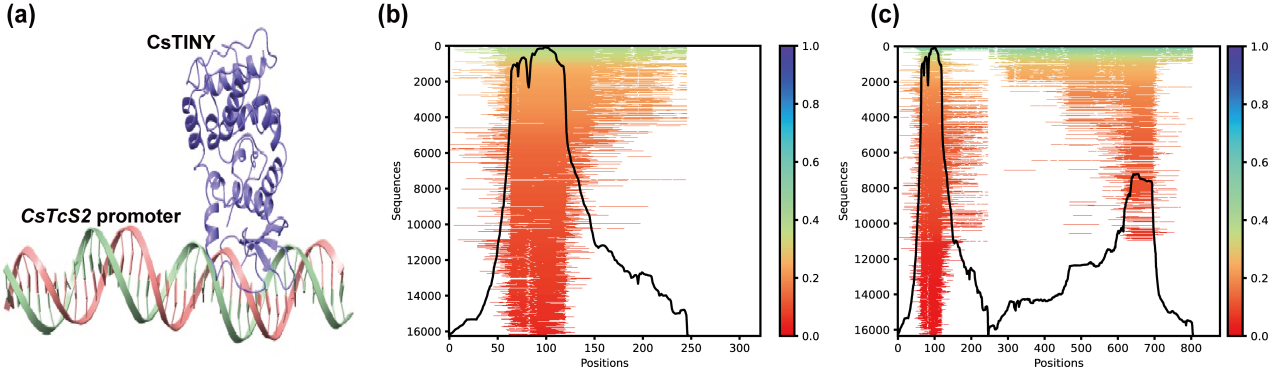


**Figure S8. Two-dimensional visualization analysis of sequence depth search and transcription factor binding prediction in gene promoter region**. (a) Structural schematic of CsTINY binding to the *CsTcS2* promoter region. The double helix represents promoter DNA, and the schematic above indicates the secondary structure of CsTINY. (b) Two-dimensional visualization analysis of the binding of a CsTINY and *CsTcS2* promoters. (c) Two-dimensional visualization analysis of the binding of CsTINY and CsWRKY33 to the *CsTcS2* promoter. The figure shows the results of sequence depth (coverage) analysis and prediction of potential DNA-protein binding hotspots in the gene promoter region. The horizontal axis represents the base position (Positions) in the genome, and the vertical axis represents the corresponding sequence (Sequences). The color gradient in the figure (from red to green) illustrates the spatial distribution of binding likelihood or binding strength. The darker the color (red), the higher the predicted binding confidence. The black contour lines at the edges describe the coverage boundaries of gene regions. The color scale on the right (from 0 to 1) corresponds to the numerical values of binding likelihood or binding probability. Areas in red have scores close to 0, while areas in green have scores close to 1, indicating potential protein-DNA binding sites in specific regions. The peaks of CsTINY appeared at 0-200 and CsWRKY33 appeared at 600-800, representing two obvious binding hot spots, which may represent the key regulatory elements of specific binding between CsTINY and CsWRKY33.


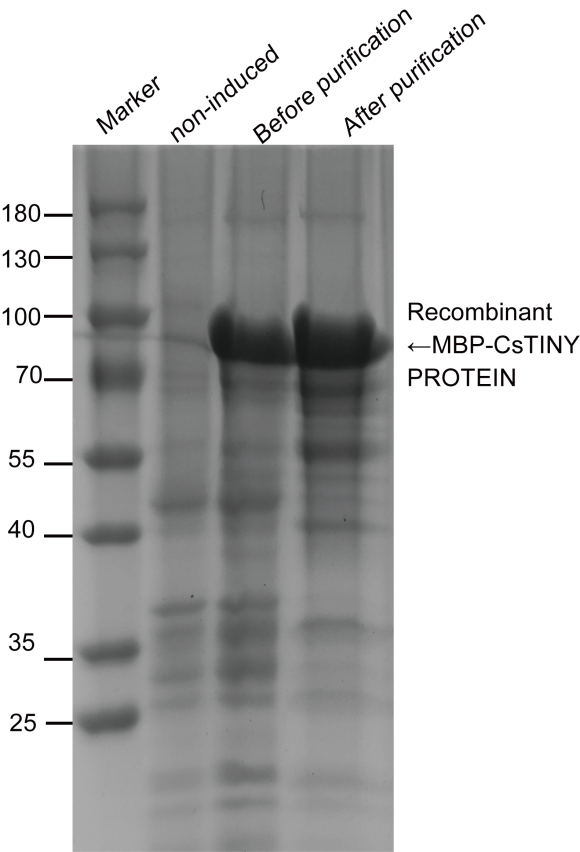


**Figure S9. SDS-PAGE analysis confirming purification of MBP-CsTINY fusion protein.**


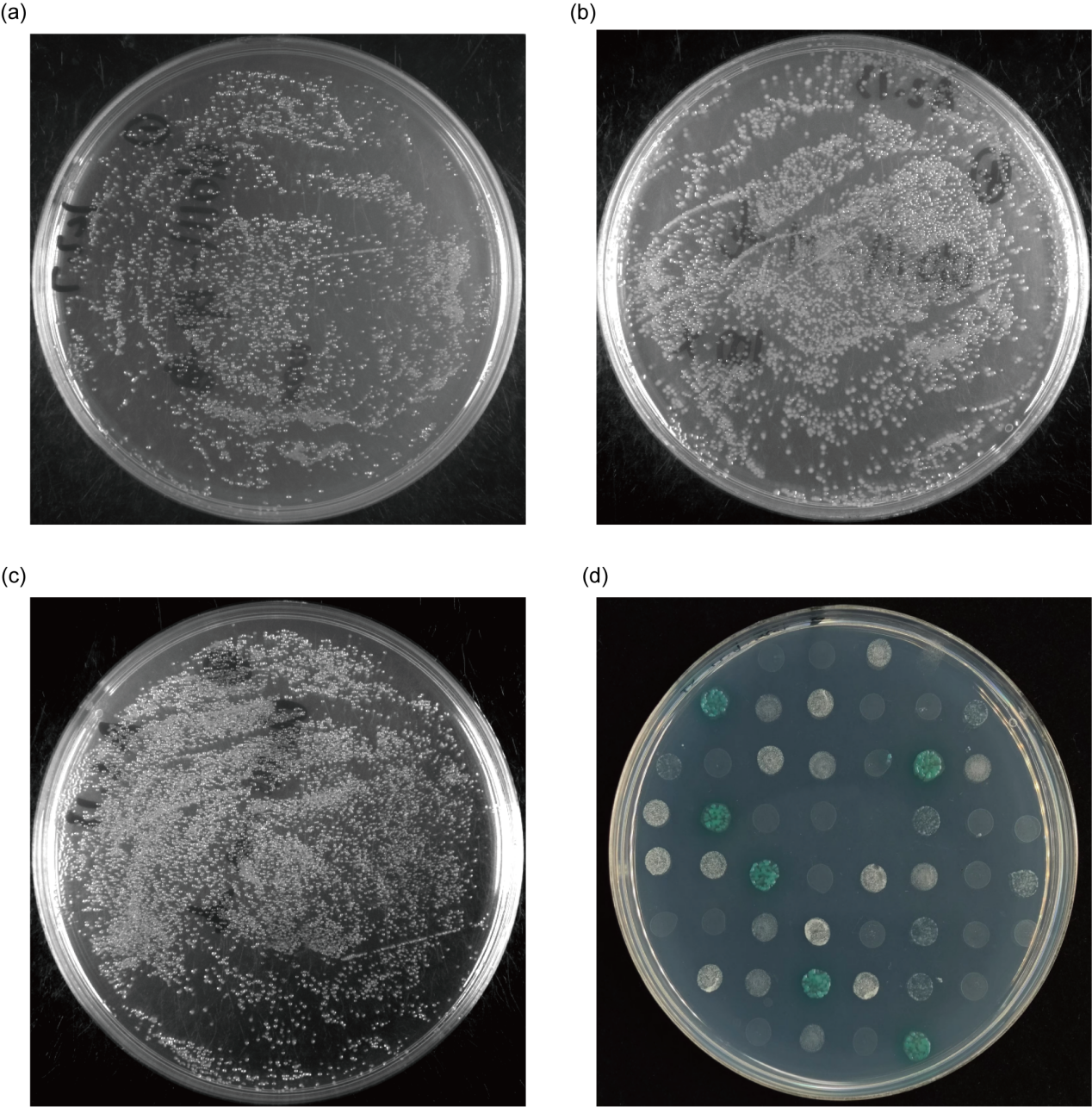


**Figure S10. Yeast double hybrid screening. To identify proteins interacting with CsTINY, we conducted a yeast two-hybrid (Y2H) screen using CsTINY as bait against a tea (*Camellia sinensis*) cDNA library.** (a) Primary yeast library capacity identification. (b). Nuclear system secondary library capacity identification. (c). Membrane system secondary library capacity identification. (d). The yeast double hybrid screening library CsTINY was used as the bait protein.


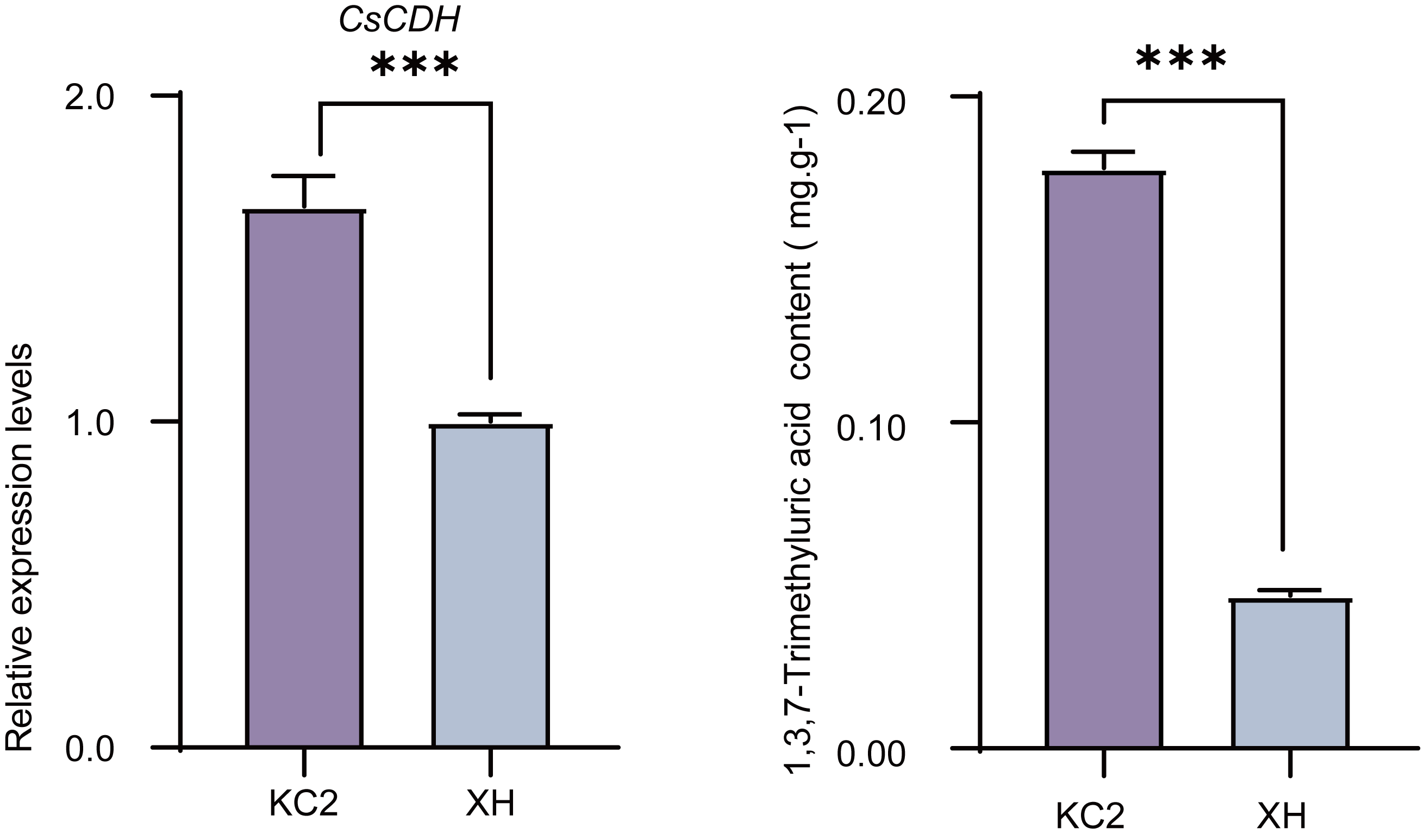


**Figure S11. Comparison of *CsCDH* expression and 1,3,7-trimethyluric acid content between the tea cultivars ‘KC2’ and ‘XH’** (***P < 0.001, two-tailed 11Student’s t-test).

**
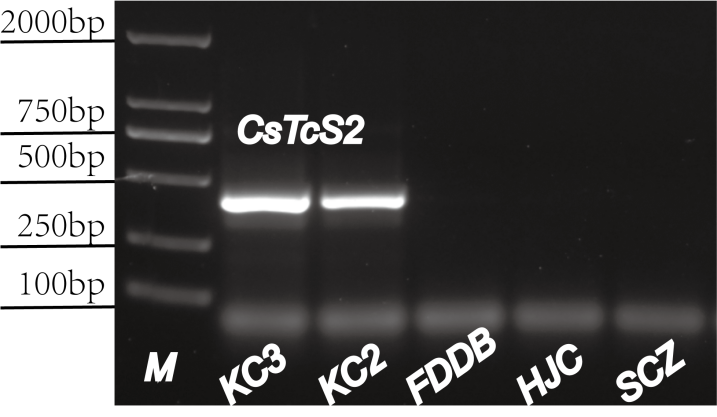
**

**Figure S12. Semi-quantitative RT-PCR analysis of *CsTcS2* expression using cDNA from theacrine-producing and non-theacrine-producing tea accessions as templates.**
